# Compromised cell competition exhausts neural stem cells pool

**DOI:** 10.1111/cpr.13710

**Published:** 2024-07-15

**Authors:** Chenxiao Li, Mengtian Zhang, Yushan Du, Shuang Liu, Da Li, Shukui Zhang, Fen Ji, Jingjing Zhang, Jianwei Jiao

**Affiliations:** ^1^ Affiliated Hospital of Guangdong Medical University & Key Laboratory of Zebrafish Model for Development and Disease of Guangdong Medical University Zhanjiang China; ^2^ Key Laboratory of Organ Regeneration and Reconstruction, Chinese Academy of Science Beijing China; ^3^ University of Chinese Academy of Sciences Beijing China; ^4^ Beijing Institute for Stem Cell and Regenerative Medicine, Institute for Stem Cell and Regeneration, Chinese Academy of Sciences Beijing China; ^5^ College of Basic Medicine, Qingdao University Qingdao China; ^6^ Jiaozuo Hospital of Traditional Chinese Medicine Henan China

## Abstract

Blood vessels play a crucial role in maintaining the stem cell niche in both tumours and developing organs. Cell competition is critical for tumour progression. We hypothesise that blood vessels may act as a regulator of this process. As a pioneer, the secretions of blood vessels regulate the intensity of cell competition, which is essential for tumour invasion and developmental organ extension. Brd4 expresses highly in endothelial cells within various tumours and is positively correlated with numerous invasive genes, making it an ideal focal point for further research on the relationship between blood vessels and cell competition. Our results indicated that the absence of endothelial Brd4 led to a reduction in neural stem cell mortality and compromised cell competition. Endothelial Brd4 regulated cell competition was dependent on Testican2. Testican2 was capable of depositing Sparc and acted as a suppressor of Sparc. Compromised cell competition resulted in the depletion of neural stem cells and accelerated brain ageing. Testican2 could rescue the run‐off of neural stem cells and accelerate the turnover rate of neurons. AD patients show compromised cell competition. Through the cloning of a point mutant of Brd4 identified in a subset of AD patients, it was demonstrated that the mutant lacked the ability to promote cell competition. This study suggests a novel approach for treating age‐related diseases by enhancing the intensity of cell competition.

## Graphical abstract

Neural stem cells with different level of Sparc step towards different fate routes, Vascular derived Testican2 regulates the level of Sparc, block of Brd4 decreased the expression of Testican2 and results in the homogenisation of neural stem cells, which is detrimental to the stemness maintaining.

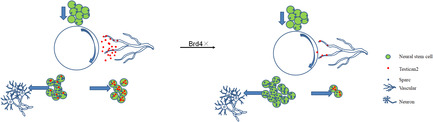

## 1 INTRODUCTION

Cell competition is a phenomenon that can be understood as an extension of Darwinian theory, wherein the fittest cells are able to survive and integrate into organs, while less fit cells are eliminated. This mechanism of quality control within cells serves to correct errors during DNA replication and optimise the selection of essential materials that make up an organism. While cell competition is commonly recognised for its role in tumour promotion and expansion, where stronger cancerous cells outcompete normal cells in their vicinity,^1^, ^2^, ^3^ it is important to note that its initial study was focused on developmental processes. Several studies conducted decades ago have demonstrated that Minute can induce cell competition in the wing disc by modulating the relative cell proliferation rate.^4^, ^5^ Subsequently, Flower was identified as a regulator of cell competition that does not affect cell proliferation rate.^6^ Additionally, cell competition has been observed in the neural system, where less fit neurons at the edge of the retina are eliminated during retina development.^7^ Furthermore, cell competition plays a role in the generation of newborn neurons during Drosophila brain regeneration following an injury.^8^ Recently, Sun discovered the presence of cell competition in the developing brain.^9^ Recent studies have indicated that compromised cell competition may lead to a shortened lifespan.^10^ However, the underlying mechanisms of this phenomenon remain unclear. Cell competition is a crucial and evolutionarily conserved process that plays a significant role in development and disease progression. It is not yet known if cell competition serves any additional functions beyond its role in determining cell death.

In addition to nutritional supplementation, emerging evidence suggests that blood vessels play a critical role in maintaining stemness in both tumours^11^, ^12^ and normal developing organs.^13^ Owing to the synchronisation of blood vessel and the extension of organs and tumours, the function of cell competition on cancer progression, we hypothesise that blood vessel may take prat in the extension of tumours and organs through cell competition. As a pioneering factor, the secrets of blood vessels modulate tumour invasion and organ development by regulating cell competition intensity.

Brd4 is belonging to the bromodomain and Extra Terminal Domain family, it functions as a chromatin reader protein that binds to acetylated histones and regulates transcription at various levels.^14^, ^15^, ^16^, ^17^, ^18^ Abnormal expression of Brd4 has been implicated in the advancement of numerous cancer types, leading to the successful use of bromodomain‐targeting antagonists in tumour therapy.^19^, ^20^, ^21^, ^22^ Through Single Cell Portal (https://singlecell.broadinstitute.org/single_cell), we found that Brd4 expressed highly in endothelial cells across multiple cancer types (Figure S1A). Through TISCH (http://tisch.comp-genomics.org/documentation/), we found a significant association between Brd4 and numerous invasive genes across various cancer types (Table S1), suggesting a potential role for Brd4 in mediating the interaction between blood vessels and cell competition. To investigate the effects of endothelial Brd4 deletion on developmental neural stem cells and potential tumour invasion, we conducted experiments using Brd4^cKO^(by breeding Brd4 flox mice with Tie2 cre mice) mice.

Our findings indicate that Brd4^cKO^ mice exhibited reduced cell death in the developing brain due to impaired cell competition. This disruption of cell competition led to the loss of neural stem cells and a decrease in the stability of stem cell maintenance. Testican2, originating from endothelial cells, serves as a primary regulator of cell competition by acting as an antagonist to Sparc. Excess Sparc diminishes the intensity of cell competition and prompts the depletion of neural stem cells, mirroring the effects of endothelial Brd4 deletion. Testican2 has the potential to replenish the neural stem cell pool in a senile mouse model. Additionally, our findings indicate a decrease in cell competition during ageing and age‐related diseases such as Alzheimer's Disease. Mutations in Brd4 may contribute to the development of Alzheimer's Disease by impairing cell competition. This research has implications for potential therapeutic interventions about cell competitive related diseases.

## 2 MATERIALS AND METHODS

### 2.1 Mice

The ICR pregnant mice used for in utero electroporation were purchase from Vital River Laboratories (Beijing, China). The Tie2‐Cre (TEK‐Cre) mice were purchased from the Shanghai Model Organism. Mice were raised under standard conditions on a 12 h light and 12 h dark cycle. and all of the animal experimental procedures were approved by the Animal Committee of Institute of Zoology, Chinese Academy of Sciences.

### 2.2 Human subjects

All dementia related data are from public resource Allen Brain Atlas.

### 2.3 Generation of Brd4^cKO^ mice

The Brd4^flox/flox^ mice were generated according to the procedures as previously described.^23^ Brd4^flox/flox^ mice were crossed with Tie2‐Cre mice to generate Brd4^cKO^ mice, genotyping PCR primers were shown in Table S4.

### 2.4 In utero electroporation

The pregnant mice were anaesthetised by sodium pentobarbital with intraperitoneal injection. Recombinant plasmid (about 1 μL at a concentration of 1.5 μg/μL) was injected into the embryonic lateral ventricle gently, embryo brains were electroporated with five 50 ms pulses at 45 V with 950 ms interval by an electroporator (BTX ECM830).^24^

### 2.5 RNA‐sequencing analysis

E14 embryo brain vascular endothelia cells of Brd4^cKO^ and Brd4(Tie2)‐f/f mice were isolated by flow sorting, total RNA of endothelia cells was extracted by Tiangen RNAprep Pure Micro Kit (# DP420), after quality control and cDNA library built, high‐throughput sequencing by Illumina HiSEq. Two thousand and five hundred platforms was followed.

### 2.6 Immunostaining

Samples were fixed by 4% paraformaldehyde for 24 h, then 30% sucrose was used for dehydration for 24 h, after sliced, brain slices were fixed by 4% paraformaldehyde for half an hour, then washed three times by PBS with 0.1% Triton X‐100, 5% bovine serum albumin was used for blocking for 1 h, then they were incubated by primary antibody overnight at 4°C, after three washes, they were incubated by secondary antibody for 1 h at room temperature.^25^ Images for immunostaining were obtained by Zeiss LSM 880.

### 2.7 Western blotting and co‐immunoprecipitation

Samples were lysed with RIPA lysis buffer supplemented with 1% PMSF and 1% protease inhibitor cocktail after ultrasonication and centrifugation, supernatants were collected for following concentration measurement, then they were denatured by boiling for 10 min, after that, protein samples were separated by 10% or 12% SDS–PAGE gel and transferred onto polyvinylidene fluoride (PVDF) membranes, then blocked by 5% BSA or milk in 0.05% PBST (PBS containing 0.02% Tween‐20) for 1 h at room temperature and incubated with primary antibody overnight at 4°C, after three washes, membranes were incubated with secondary antibody for 1 h at room temperature, after three washes, membranes were scanned by Image Studio Ver 5.2 software.^26^ The control is β‐Actin or Gapdh.

For co‐immunoprecipitation (co‐IP), protein samples were lysed and supernatants were collected, then incubated with anti‐HA‐tag (MBL) or anti‐Flag‐tag magnetic beads (MBL), after three washes, bound proteins were used for western blotting (WB).

### 2.8 BrdU labelling

10 W C57 male mice were intraperitoneally injected with d‐galactose (180 mg/kg) daily for 4 months. Then proteins (600 ng) were Stereotactic injected into hippocampus. After 7 days, mice were intraperitoneally injected with BrdU (50 mg/kg) for four times within 12 h and harvested after 24 h.

### 2.9 Real‐time PCR

Total RNA was extracted by TRIzol (Invitrogen, 15,596) method and reverse transcribed into cDNA by FastQuant RT Kit (Tiangen). Real‐time PCR (RT‐PCR) were conducted by SYBR qPCR master mix (Tiangen) using ABI7500 real‐time PCR system (Applied Biosystems). 𝛽‐Actin was seen as endogenous control for normalisation. Related primers were shown in Table S4.

### 2.10 Antibodies

The following primary antibodies and dilutions were used for immunostaining and WB: Brd4 (Abcam ab128874, Rabbit, 1:200); Tbr2(Abcam, ab23345, Rabbit, 1:1000); Sox2 (Cell Signaling Technology, 3728 s, Rabbit, 1:1000); GFAP (Sigma, G6171, Mouse, 1:1000); cleaved Casp3 (Cell Signaling Technology, 9664 s, Rabbit, 1:500); biotinylated IsolectinB4 (Vector Laboratories, B‐1205, 1:600); HA (Cell Signaling Technology, Rabbit, 1:1000); Flag (Sigma, F7425, Mouse, 1:2000); 𝛽‐Actin (Proteintech, 20,536‐1‐AP, Rabbit, 1:10000); 𝛽‐Actin (Proteintech; 60,008‐1‐Ig Mouse,1:2000); Sparc (Abcam, ab225716, Rabbit, 1:1000), IgG (Bioss, bs‐0295p; Rabbit, 1:1000).

Secondary antibodies: for immunostaining: DAPI (2 mg/mL; Sigma; D9542); Alexa Fluor 488, Cy3, or Cy5 (Jackson ImmunoResearch, 1:1000); for WB: Donkey AntiIgG (LI‐COR Biosciences 800CW; Rabbit, 1:1000); Donkey Anti‐IgG (LI‐COR Biosciences 800CW; Mouse, 1:1000); Donkey anti‐IgG (LICOR Biosciences 680LT; Rabbit, 1:1000); and Donkey anti‐IgG (LICOR Biosciences 680LT; Mouse, 1:1000).

### 2.11 Statistical analysis

Data were present as mean ± SEM. Data normality of distribution were assessed before statistical analysis. The comparisons between two groups were analysed by unpaired two‐tailed Student's *t* test or Wilcoxon Test. The comparisons among more than two groups were analysed by ANOVA. All data were analysed by GraphPad Prism 6 software. n.s., not significant, *p < 0.05; **p < 0.01; ***p < 0.001.

## 3 RESULTS

### 3.1 Brd4 is involved in the competition of neural stem cells

**FIGURE 1:**
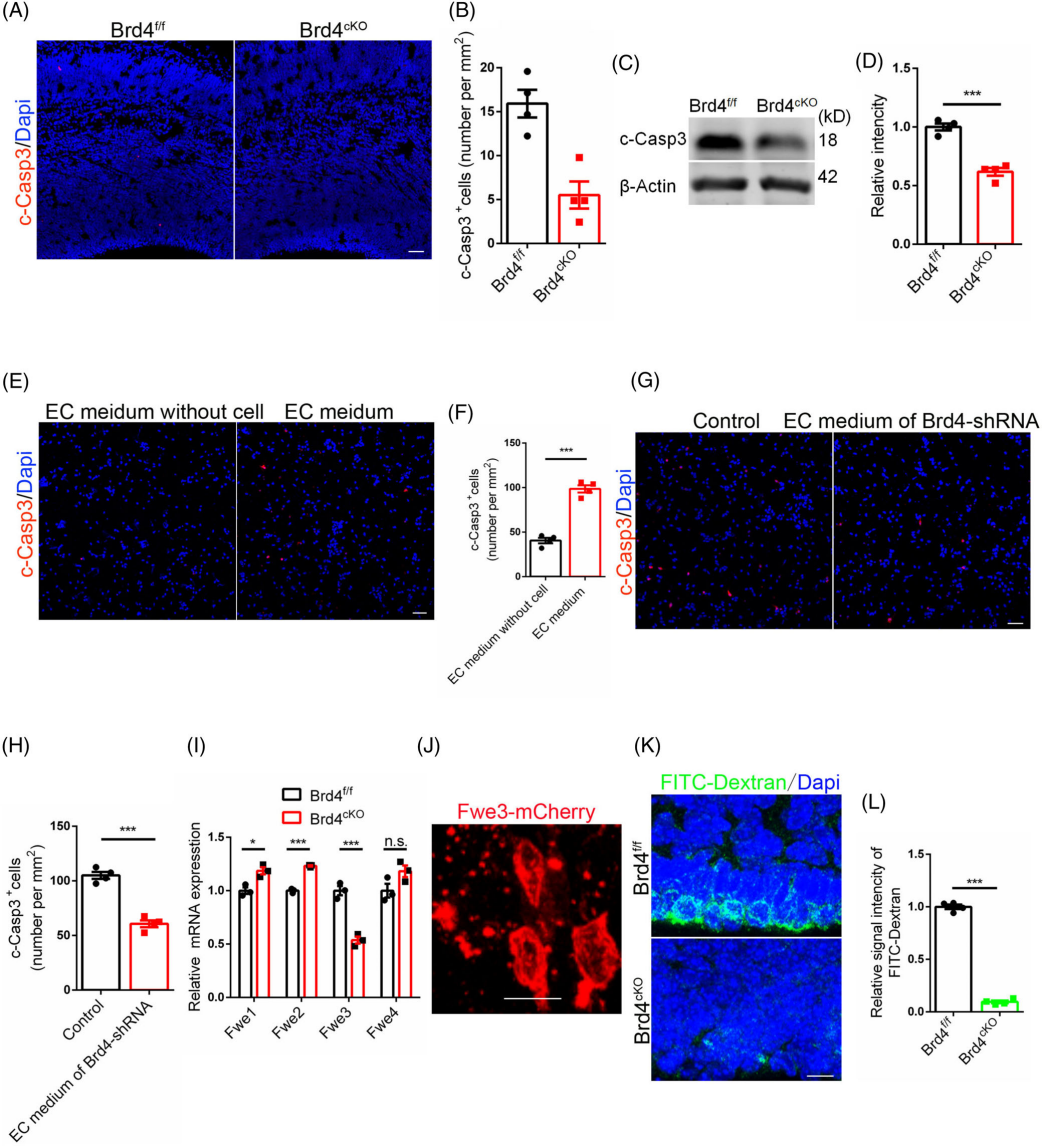
Brd4 regulates the cell competition of neural stem cells during brain development. (A) Immunofluorescent staining for c‐Casp3 with mice brains at E15.5. Scale bar, 50 μm. (B) Statistic analysis of c‐Casp3 positive cells, *n* = 4. (C) WB detection for c‐Casp3 with mice cerebral cortex at E14. (D) Statistic analysis of relative intensity of c‐Casp3 in WB detection, *n* = 4. (E) Immunofluorescent staining for c‐Casp3 with primary neural stem cells after addition of endothelial medium. Scale bar, 50 μm. (F) Statistic analysis of c‐Casp3 positive cells, *n* = 4. (G) Immunofluorescent staining for c‐Casp3 with primary neural stem cells after addition of endothelial medium (Brd4 knock‐down). Scale bar, 50 μm. (H) Statistic analysis of c‐Casp3 positive cells, *n* = 4. (I) Flower isoforms detection by RT‐PCR using mice cerebral cortex at E14, *n* = 3. (J) Distribution of Fwe3‐mCherry in neural stem cells (E13.5–15). (K) Phagocytosis ability detection of mice at P0 using FITC‐Dextran.(L) Statistic analysis of relative signal intensity of FITC‐Dextran, *n* = 4.

Endothelial Brd4 conditional knockout mice were obtained by generating Brd4 flox mice, as shown in Figure S1B, followed by crossing with Tie2 cre mice. High knockout efficiency was confirmed through WB detection and immunofluorescent staining using primary brain vascular cells (Figure S1C–E). In vivo studies further supported the high knockout efficiency (Figure S1F). Notably, Brd4^cKO^ mice exhibited a lower number of cleaved Casp3 (c‐Casp3) positive cells in the developing brain (Figure 1A,B). Consistent results were also observed in WB detection (Figure 1C,D). Similar results were found at the mice brain at P0 (Figure S1G,H). Given the role of blood vessels in stemness maintenance, further investigation is warranted to explore its potential secretory functions. We added medium had cultured bEnd.3 (brain‐derived Endothelial cells. 3) or not into medium of neural stem cells respectively. Data said that the former could yield more deaths among neural stem cells in vitro (Figure 1E,F, Figure S1I,J). Furthermore, knocking Brd4 down led to a decrease in cell death (Figure 1G,H, Figure S1K,L), while overexpression of Brd4 resulted in an increase in cell death (Figure 9E–H). While we found that not all but only a small portion of cells died, this said that the secreted factors were not toxic. The necrosis signalling pathway in Brd4^cKO^ mice was subsequently identified, with Tnfα detection revealing comparable levels between the two groups (Figure S1M,N). The canonical cell competitive marker protein Flower isoforms in Brd4^cKO^ mice were then examined, revealing a significant decrease in the mRNA level of Fwe3 (Figure 1I). Knock‐down of Brd4 resulted in a reduction of Fwe3 mRNA levels induced by endothelial cultural supernatant (Figure S2A). Subsequent investigation into whether Fwe3 could induce cell death via in utero electroporation (IUE) demonstrated a contrast to its normal expression at the cytomembrane, numerous red signals were observed to be dispersed or clustered, indicating cell apoptosis (Figure 1J). The role of cell competition in the induction of cell death by Fwe3 was investigated. To address this inquiry, Fwe3‐mCherry was overexpressed in neural stem cells in vitro, revealing that Fwe3 triggered cell death only in the presence of genetically distinct neural stem cells; individual cells overexpressing Fwe3‐mCherry did not undergo apoptosis (Figure S2B). These findings suggest that the homogenisation of neural stem cells resulting from the absence of Fwe3 attenuated the competitive interactions between cells and reduced the incidence of cell death during brain development.

**FIGURE 2:**
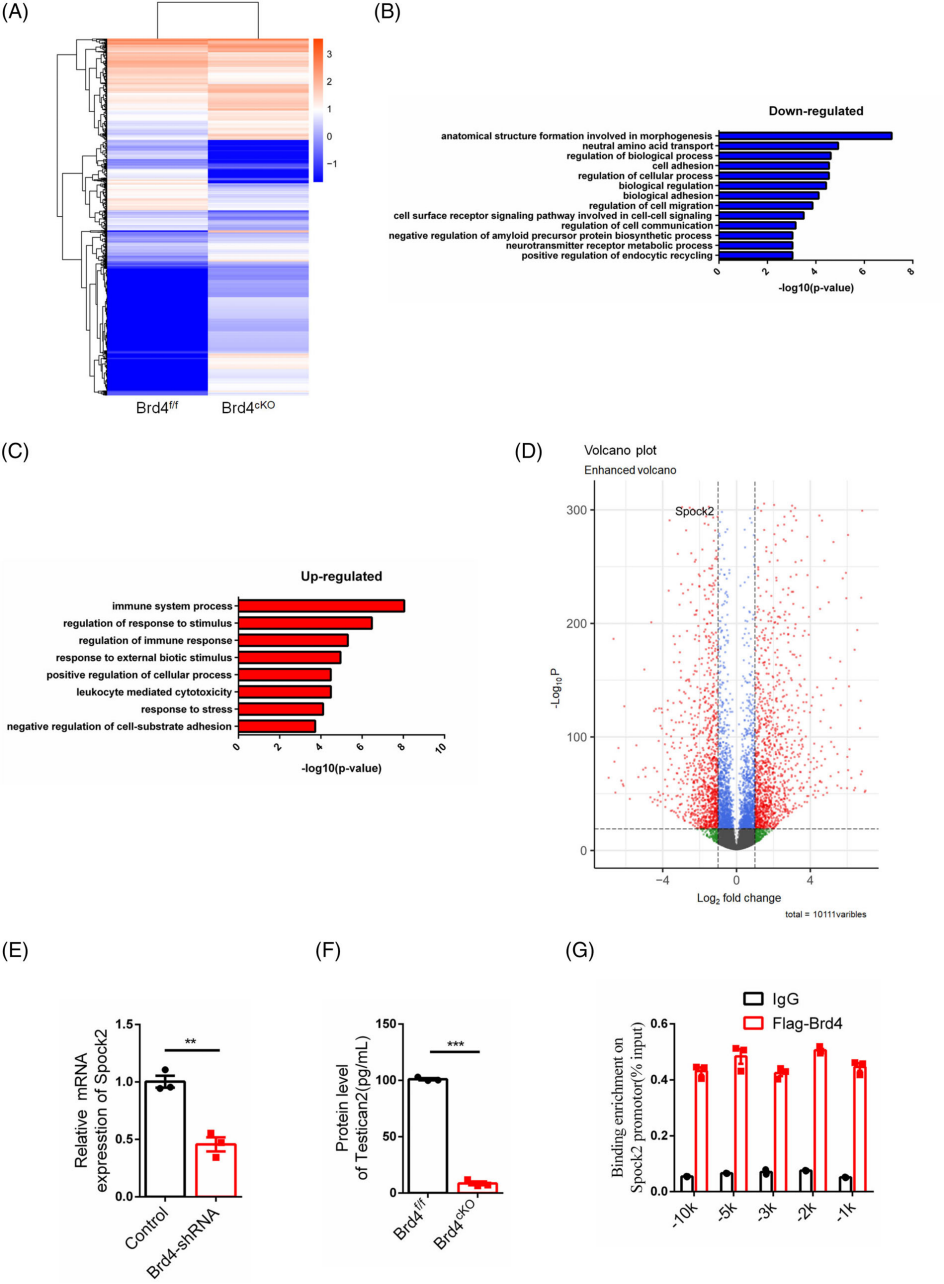
RNA‐seq analysis of mice brain vascular endothelial cells. (A) Heap map of RNA sequencing data of brain vascular endothelial cells from E14.5 mice. (B, C) Down‐regulated and up‐regulated signalling pathways by Gene ontology analysis. (D) Distribution of down‐regulated and up‐regulated genes showed by Volcano plots. (E) The level of Spock2 detection by RT‐PCR in bEnd.3 cells after Brd4 knock‐down, *n* = 3. (F) Detection of the level of Testican2 with cultured supernatant of brain vascular endothelial cells taken from Brd4^cKO^ mice and their littermates at E14.5 by ELISA, *n* = 3. (G) Detection of binding potential of Brd4 on the promotor of Spock2, *n* = 3.

Phagocytosis was identified in neural stem cells,^9^, ^27^ indicating that dominant cells engulfed neighbouring cells. The inhibition of cell competition resulted in a decrease in phagocytosis. To further investigate the weakened cell competition in Brd4^cKO^ mice, FITC‐dextran was injected into the ventricle to assess the phagocytic ability of neural stem cells. The results demonstrated reduced phagocytosis in Brd4^cKO^ mice (Figure 1K,L), which also evidenced by decreased expression of the canonical engulfment gene Elmo1 (Figure S2C). These findings suggest that the absence of endothelial Brd4 may play a role in decreased cell deaths by weakening the intensity of cell competition.

### 3.2 Brd4 regulates the competition of neural stem cells through Spock2

In order to identify the primary mediator, endothelial cells were isolated from brain blood vessels and subjected to RNA sequencing. The results indicated that the deletion of Brd4 resulted in alterations in gene expression patterns (Figure 2A). Subsequent gene ontology analysis revealed that the suppressed signalling pathways pertained to cell adhesion, migration, communication, and cell surface receptor‐mediated cell–cell signalling in Brd4^cKO^ mice (Figure 2B), indicative of diminished cell competitiveness. Conversely, the enhanced signalling pathways were associated with responses to external stimuli and stress (Figure 2C). Genes encoding secretory proteins were selected from the top differentially expressed genes (Figure 2D), and subsequently knocked them down in endothelial cells. The supernatant from these cells was collected and added to the medium of neural stem cells. The results indicated that knockdown of Spock2, which encodes the secretory protein Testican2, exhibited similar effects to knockdown of Brd4 (Figure S3A). Additionally, Brd4 knockdown led to a significant reduction in Spock2 mRNA levels (Figure 2E), enzyme linked immunosorbent assay (ELISA) analysis revealed lower levels of Testican2 protein in Brd4^cKO^ mice (Figure 2F), and chromatin immunoprecipitation assay (CHIP) assays demonstrated that Brd4 could bind to the promoter region of Spock2 (Figure 2G). Due to the presence of two bromodomains in Brd4 that bind to distinct downstream targets, specific antagonists have been developed for each domain. In order to explore the therapeutic potential of Brd4, we individually mutated the binding sites of the two bromodomains and utilised the resulting mutants in CHIP assays. Our findings indicate that mutating the binding site of the first bromodomain disrupted the interaction between Brd4 and the promoter of Spock2 (Figure S3B), whereas mutating the binding site of the second bromodomain had no effect on this interaction (Figure S3C). These data provided a more focused selection criteria for the treatment of diseases related to cell competition.

### 3.3 Spock2 regulates the competition of neural stem cells through Sparc

**FIGURE 3:**
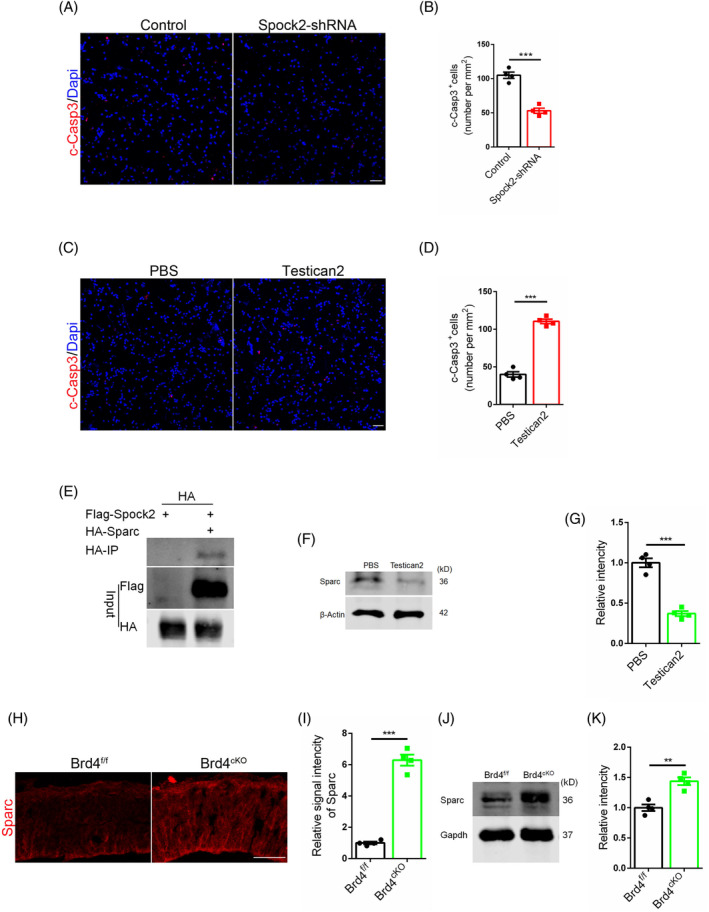
Spock2 mediates the cell competition of neural stem cells by regulating the level of Sparc. (A) Immunofluorescent staining for c‐Casp3 with primary neural stem cells after add‐in of EC medium (Spock2 knock‐down). Scale bar, 50 μm. (B, D) Statistic analysis of c‐Casp3 positive cells, *n* = 4. (C) Immunofluorescent staining for c‐Casp3 with primary neural stem cells after Testican2 treatment (150 ng/mL). Scale bar, 50 μm. (E) Contraction identification between Spock2 and Sparc using co‐IP. (F) Effect of Testican2 on the protein level of Sparc in neural stem cells. (G) Statistic analysis of relative intensity of Sparc in WB detection, *n* = 4. (H) Sparc staining of mice brain at P0. Scale bar, 50 μm. (I) Statistic analysis of relative signal intensity of Sparc, *n* = 4. (J) WB detection of Sparc using mice cerebral cortex at P0. (K) Statistic analysis of relative intensity of Sparc in WB detection, *n* = 4.

Spock2 is belonging to the SPARC/osteonectin (FS‐EC), CWCV (TY), and Kazal‐like family.^28^ Existing researches predominantly focus on tumour^29^, ^30^ and lung diseases,^31^, ^32^ with limited investigation into its role in neurological research, the only one is that it inhibits neurite extension.^33^ However, its function in brain development remains largely unexplored. Our study showed that knocking down endothelial Spock2 reduced neural stem cell deaths induced by EC medium (Figure 3A,B). While the addition of Testican2 protein increased stem cell deaths (Figure 3C,D). Our research has identified Spock2 as the primary target of Brd4, while Spock2 is not a recognised cell competitive gene. This raises the question of how Spock2 facilitates the interaction between Brd4 and cell competition. Spock2 is known to mediate the degradation of its family members,^34^ leading us to focus on Sparc, a well‐known regulator of cell competition. Sparc has been shown to protect outcompeted cells from death by inhibiting caspase activation in Drosophila development.^35^ Through co‐IP and WB assays, we demonstrated that Spock2 could bind to Sparc and decrease its protein levels. (Figure 3E–G). Brd4^cKO^ mice exhibited elevated Sparc protein levels (Figure 3H‐K). Sparc could also mitigate the mortality of neural stem cells induced by the introduction of endothelial cell medium with Brd4 overexpression in vitro (Figure 9E,F).

### 3.4 Sparc determines the fate transition of neural stem cells

**FIGURE 4:**
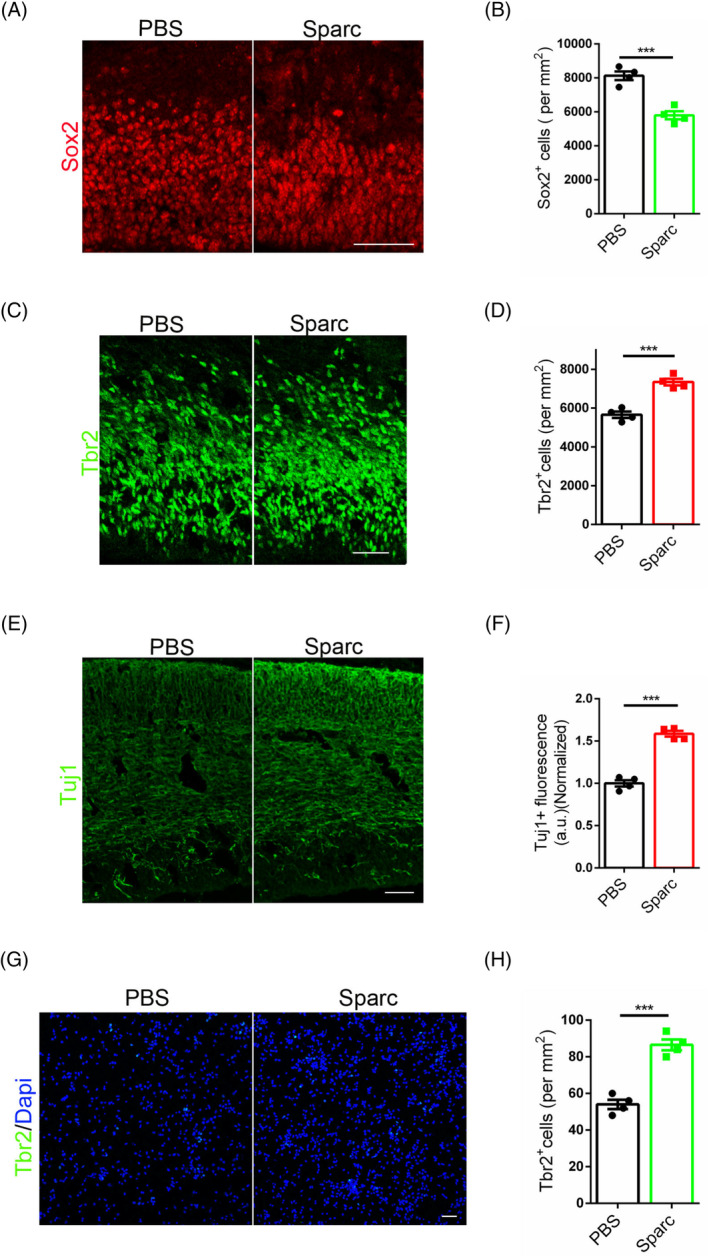
Sparc is the fate decider of neural stem cells. (A) Immunofluorescent staining for Sox2 with mice brains at E15 after Sparc treatment (before 24 h). Scale bar, 50 μm. (B) Statistic analysis of Sox2 positive cells, *n* = 4. (C) Immunofluorescent staining for Tbr2 with mice brains at E15 after Sparc treatment (before 24 h). Scale bar, 50 μm. (D) Statistic analysis of Tbr2 positive cells, *n* = 4. (E) Immunofluorescent staining for Tuj1 with mice brains at E15 after Sparc treatment (before 24 h). Scale bar, 50 μm. (F) Statistic analysis of Fluorescence intensity of Tuj1, *n* = 4. (G) Immunofluorescent staining for Tbr2 with neural stem cells after Sparc treatment. Scale bar, 50 μm. (H) Statistic analysis of Tbr2 positive cells, *n* = 4.

Despite the potential induction of cell death through cell competition during development, only a minority of cells ultimately succumb. When considering the criteria of cell survival, it may appear that cell competition is nonessential. However, studies have shown that compromised cell competition can have negative consequences. This raises the question of whether cell competition serves additional functions, particularly in the context of brain development. In contrast to the extensive body of research on tumours, there has been relatively less focus on neurological research pertaining to Sparc, despite its abundant expression in the brain. Sparc plays a critical role in synapse formation^36^, ^37^, ^38^, ^39^, ^40^ and the development of certain neurological diseases.^41^, ^42^, ^43^ The potential involvement of Sparc in the determination of neural stem cell fate remains unknown. To address this question, Sparc protein was injected into the ventricles of mice at embryonic day 14 (E14) and the brains were harvested after 24 h. Immunostaining for Sox2 revealed a significant decrease in the number of Sox2 positive cells following Sparc injection (Figure 4A,B), while staining for Tbr2 showed an increase in the number of Tbr2 positive cells (Figure 4C,D). The results of Tuj1 staining indicated that Sparc injection produced a more pronounced differentiation signal compared to the control group (Figure 4E,F). In vitro experiments further demonstrated that an excess of Sparc decreased the numbers of neural stem cells (Figure 4G,H). To further investigate the association between Sparc and the maintenance of stemness, neural stem cells were treated with the Sparc antagonist Atorvastatin. The findings revealed that Atorvastatin reduced the numbers of neural stem cells as evidenced by both RT‐PCR and WB analysis (Figure S4A–C).

### 3.5 Compromised cell competition induced the run‐off of neural stem cells

**FIGURE 5:**
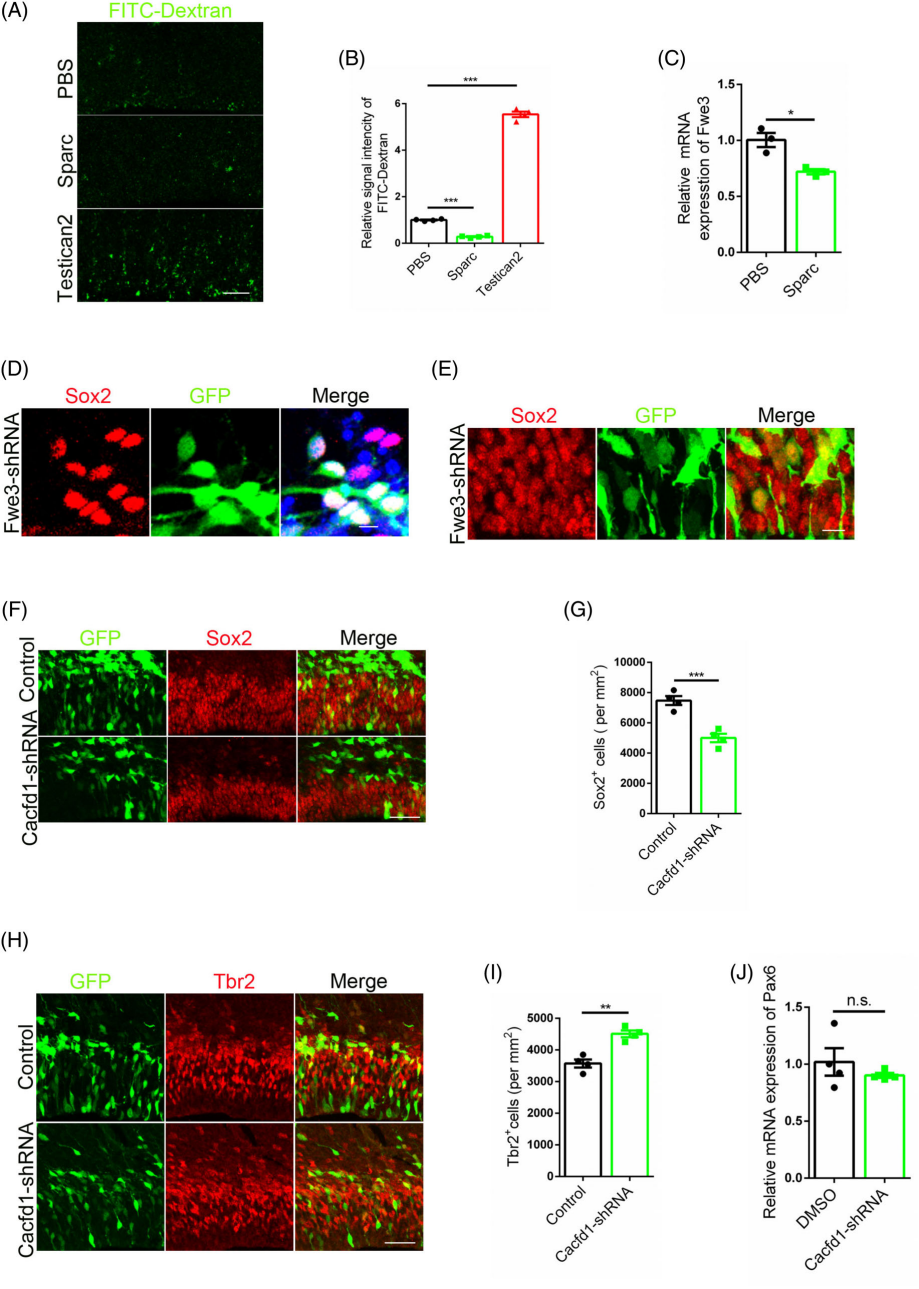
Compromised cell competition induces the run‐off of neural stem cells. (A) Phagocytosis ability detection using FITC‐Dextran after the treatment of Sparc or Testican2 (before 24 h), Scale bar, 10 μm. (B) Statistic analysis of relative signal intensity of FITC‐Dextran, *n* = 4. (C) Detection of the effects of Sparc surplus on the expression of Fwe3 among neural stem cells in vitro by RT‐PCR, *n* = 3. (D) Effect of Fwe3 knock‐down on the expression of Sox2 in primary neural stem cells. Scale bar, 10 μm. (E) Effect of Fwe3 knock‐down on the expression of Sox2 among neural stem cells by IUE. Scale bar, 10 μm. (F) Sox2 staining with mice brain after Cacfd1 knock‐down (E13‐14). Scale bar, 50 μm. (G) Statistic analysis of Sox2 positive cells, *n* = 4. (H) Tbr2 staining with mice brain after Cacfd1 knock‐down (E13‐14). Scale bar, 50 μm. (I) Statistic analysis of Tbr2 positive cells, *n* = 4. (J) RT‐PCR analysis of Pax6 using neural stem cells after Cacfd1 knockdown (Atorvastatin treatment), *n* = 4.

Our study has elucidated the role of Sparc in regulating the fate determination of neural stem cells, prompting the question of whether this process is dependent on cell competition. In addressing the inquiry, phagocytosis ability assays were carried out on neural stem cells, with findings indicating that an excess of Sparc (protein injection) attenuated the phagocytosis capability of neural stem cells, while Testican2 enhanced this ability (Figure 5A,B). RT‐PCR analysis demonstrated that Sparc surplus led to a reduction in the expression of Fwe3 in vitro (Figure 5C). To elucidate the association between the down‐regulation of Fwe3 and the differentiation of neural stem cells, Fwe3 was silenced in neural stem cells and Sox2 staining was performed. Results revealed that the shortage of Fwe3 did not result in the loss of stemness in neural stem cells in both in vitro and in vivo settings (Figure 5D,E). We subsequently performed knockdown of Cacfd1 to homogenise neural stem cells, which revealed that homogenisation led to a reduction in stemness (Figure 5F,G) and an increase in differentiation of neural stem cells (Figure 5H,I). Knockdown of Cacfd1 also diminished the ability to maintain stemness of Atorvastatin (Figure 5J). These findings suggest that the maintenance of stemness requires heterogeneity, as opposed to homogenisation which results in the depletion of neural stem cells.

### 3.6 Cell competition is critical for the stemness maintaining both at developmental stage and steady state

**FIGURE 6:**
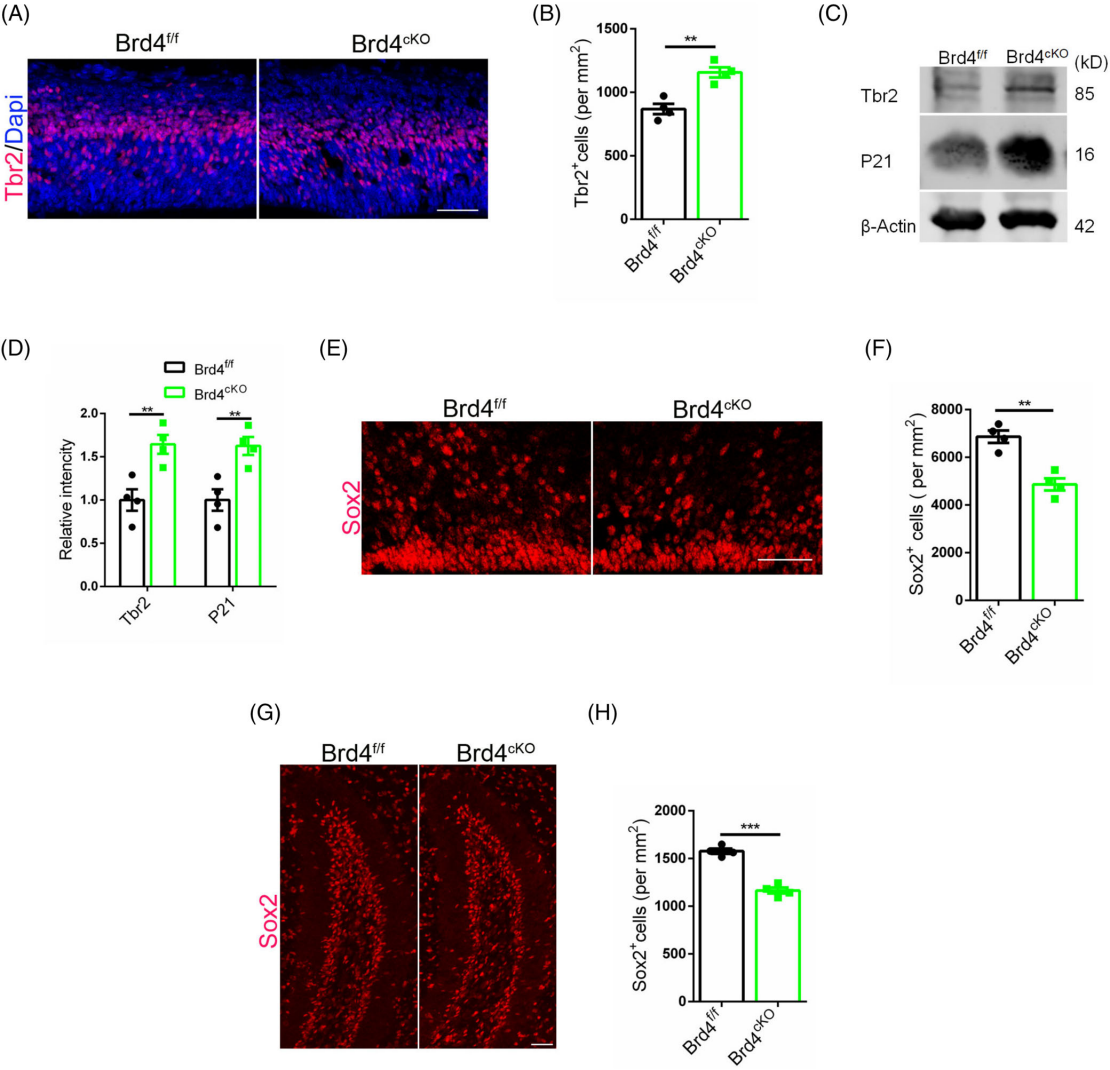
Brd4^cKO^ mice show the run‐off of neural stem cells at different periods. (A) Immunofluorescent staining for Tbr2 with mice brain at E13. Scale bar, 50 μm. (B) Statistic analysis of Tbr2 positive cells, *n* = 4. (C) WB detection for P21 and Tbr2 with mice cerebral cortex at E13. (D) Statistic analysis of relative intensity of P21 and Tbr2 in WB detection, *n* = 4. (E) Sox2 staining with mice brain at P0. Scale bar, 10 μm. (F) Statistic analysis of Sox2 positive cells, *n* = 4. (G) Sox2 staining with mice brain at P14. Scale bar, 10 μm. (H) Statistic analysis of Sox2 positive cells, *n* = 4.

**FIGURE 7:**
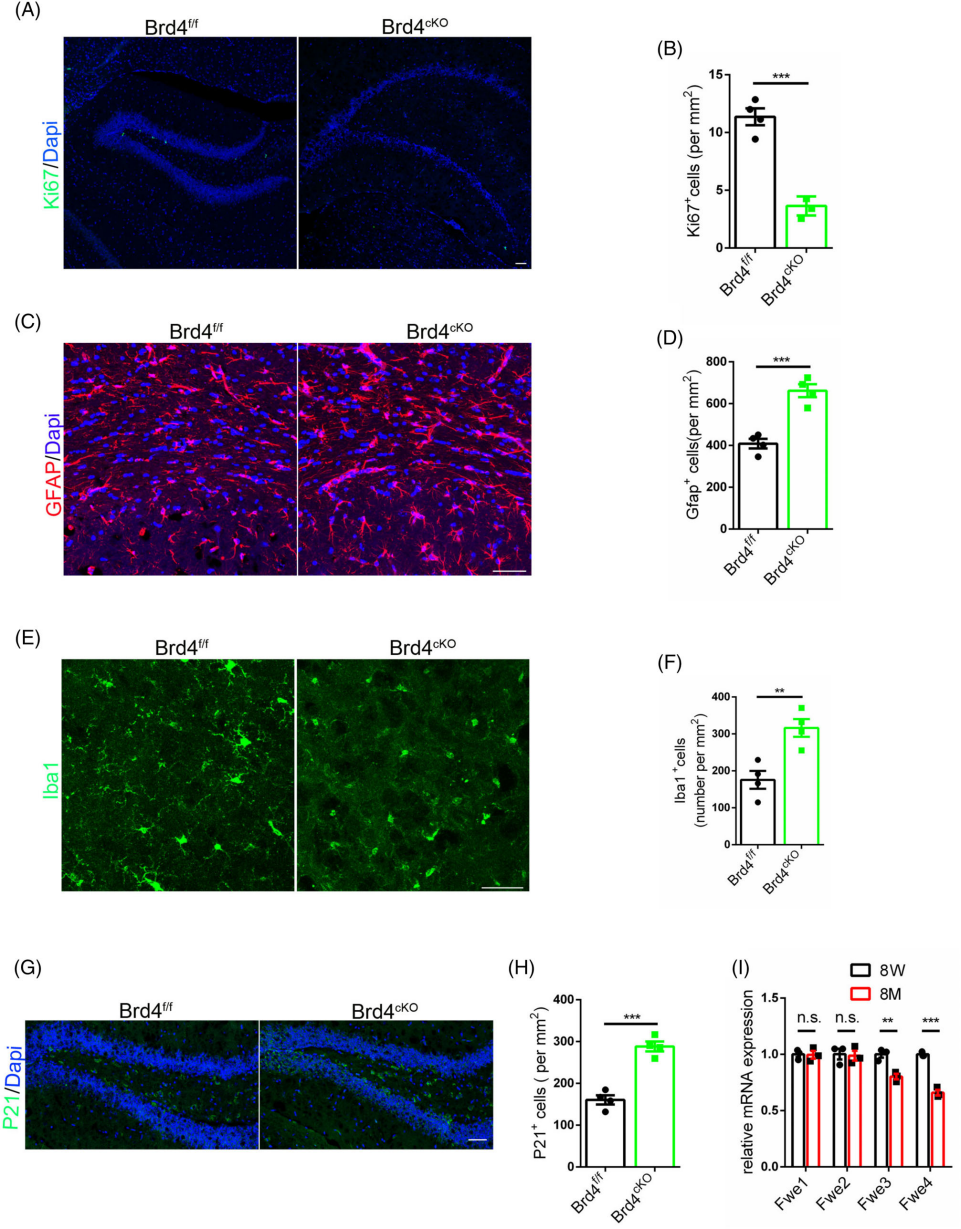
Compromised cell competition accelerating ageing. (A) Immunofluorescent staining for Ki67 with mice brain at 15 months old. (B) Statistic analysis of Ki67 positive cells, *n* = 4. (C) Immunofluorescent staining for GFAP with mice brains at 15 months old. (D) Statistic analysis of GFAP positive cells, *n* = 4. (E) Immunofluorescent staining for Iba1 with mice brains at 15 months old. (F) Statistic analysis of Iba1 positive cells, *n* = 4. (G) Immunofluorescent staining for P21 with mice brains at 15 months old. (H) Statistic analysis of P21 positive cells, *n* = 4. (I) RT‐PCR detection of Flower isoforms using cerebral cortex taken from 8 weeks mice and 8 months mice, *n* = 3. (Scale bar, 50 μm).

Our study has elucidated the correlation between compromised cell competition and the depletion of neural stem cells, while it remains to be determined if this phenomenon is applicable to Brd4^cKO^ mice. To address this question, we conducted Tbr2 staining on mouse brain at E13. The results indicated a higher number of Tbr2 positive cells in Brd4^cKO^ mice (Figure 6A,B), which was also confirmed by WB results (Figure 6C,D). Interestingly, elevated levels of P21 were observed in Brd4^cKO^ mice, suggesting a correlation between impaired cell competition and reduced cellular quality (Figure 6C,D). Additionally, Sox2 staining of mice brains at P0 demonstrated a lower presence of Sox2 positive cells in the ventricular zone of Brd4^cKO^ mice (Figure 7E,F). Furthermore, a decrease in neural stem cells was detected in the hippocampus of Brd4^cKO^ mice at P14 (Figure 6G,H), with the deficit worsening by 5 weeks of age (Figure S5A,B), indicating a critical role of cell competition on both the development and maintaining of neural stem cells.

### 3.7 Compromised cell competition results in faster brain ageing

The observation of decreased neural stem cell turnover in elderly individuals and AD patients raises the question of whether this phenomenon may contribute to the development of age‐related diseases. To investigate this, we sacrificed Brd4^cKO^ mice and their littermates when they were 15 months old. Results revealed that Brd4^cKO^ mice exhibited greater signs of senescence, as evidenced by reduced numbers of neural stem cells (Figure 7A,B) and intermediate progenitor cells (Figure S6A, B) in the hippocampus. Utilising the Single Cell Portal, we confirmed the expression of Brd4 in endothelial cells within the hippocampus (Figure S6C). Additionally, Brd4^cKO^ mice displayed an increased presence of astrocytes and a more active state, as indicated by more branching (Figure 7C,D). Iba1 staining revealed an increased presence of microglia with a more active phenotype, characterised by reduced branching (Figure 7E,F) in Brd4^cKO^ mice compared to their littermates, indicating a heightened inflammatory state. Additionally, P21 staining for senescent markers demonstrated a greater degree of cellular senescence in Brd4^cKO^ mice (Figure 7G,H; Figure S6D,E). Analysis of mRNA levels of different Flower isoforms in the cortex of mice at 8 weeks (8 W) and 8 months (8 M) of age showed a decrease in both Fwe3 and Fwe4 in the older mice, suggesting a gradual decline in cell competitive ability with ageing (Figure 7I).

### 3.8 Testican2 accelerates the turnover rate of neural stem cells

**FIGURE 8:**
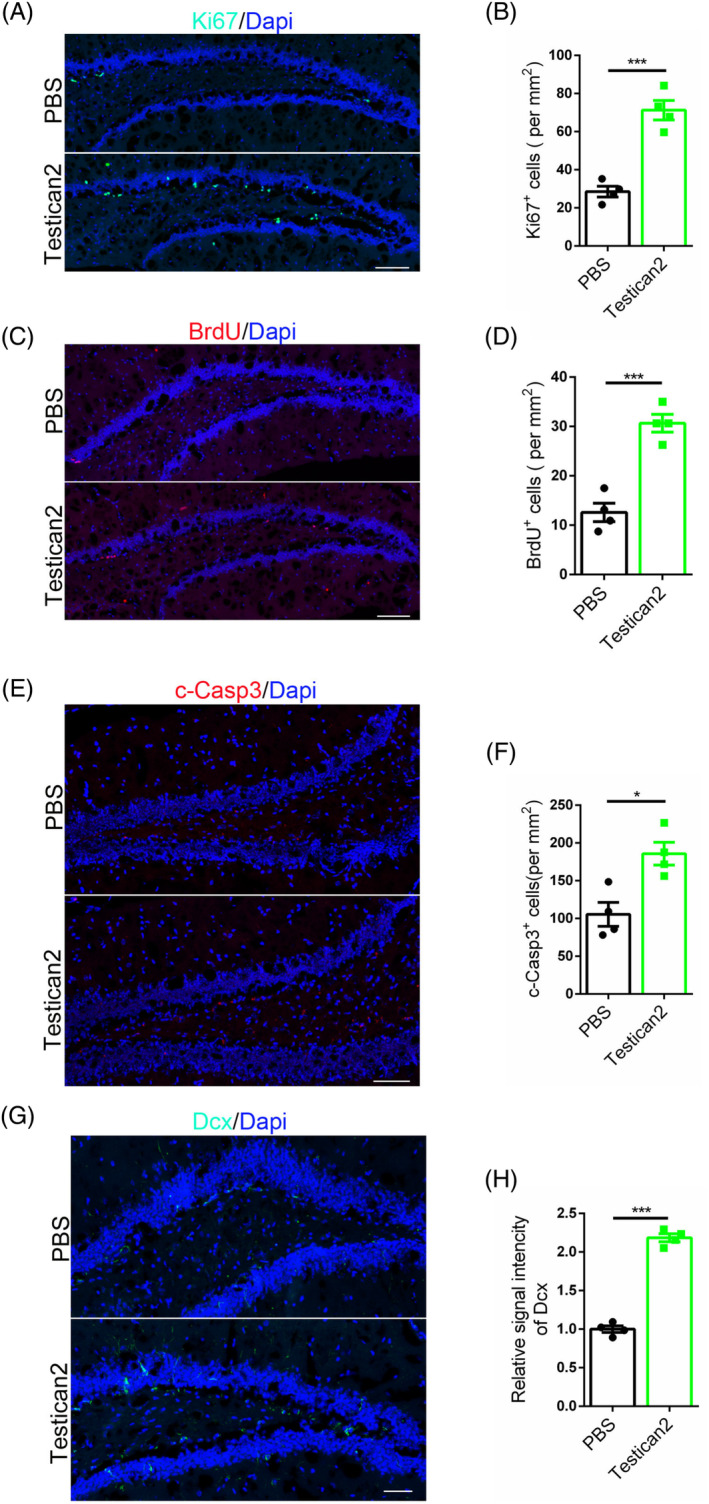
Strengthened cell competition restore the pool of neural stem cells in senile mice model. (A) Immunofluorescent staining for Ki67 with mice brain. Scale bar, 100 μm. (B) Statistic analysis of Ki67 positive cells, *n* = 4. (C) Immunofluorescent staining for BrdU with mice brain. Scale bar, 100 μm. (D) Statistic analysis of BrdU positive cells, *n* = 4. (E) Immunofluorescent staining for c‐Casp3 with mice brain. Scale bar, 50 μm. (F) Statistic analysis of c‐Casp3 positive cells, *n* = 4. (G) Immunofluorescent staining for Dcx with mice brain. Scale bar, 50 μm. (H) Statistic analysis of relative signal intensity of Dcx, *n* = 4.

Given these findings on cell competition in ageing, further investigation is warranted to determine if enhancing the intensity of cell competition could potentially delay disease progression. In this study, a senile mice model was generated through d‐galactose and subsequent stereotactic injection at the hippocampus was used to assess the effect of Testican2. Ki67 staining was utilised to assess the effects of Testican2 on neural stem cells, revealing a decrease in the run‐off of these cells in senile mice models (Figure 8A,B). Additionally, BrdU labelling demonstrated an increase in labelled cells following Testican2 treatment (Figure 8C,D). To further investigate the observed increase in neural stem cells, c‐Casp3 staining was conducted to assess cell competition, showing an increase in cell deaths in the hippocampus with Testican2 treatment (Figure 8E,F). Furthermore, Dcx staining indicated an increase in neurogenesis in the hippocampus (Figure 8G,H). This finding suggests that Testican2 accelerates the turnover rate of neural stem cells by eliminating weaker neurons and replacing them with new, healthy neurons. It is interesting that Atorvastatin also decreased the run‐off of neural stem cells detected by both RT‐PCR (Figure S7A) and WB (Figure S7B,C), WB results consolidated its powerful role on antagonising Sparc (Figure S7D,E).

### 3.9 Brd4 is negatively correlated with dementia

**FIGURE 9:**
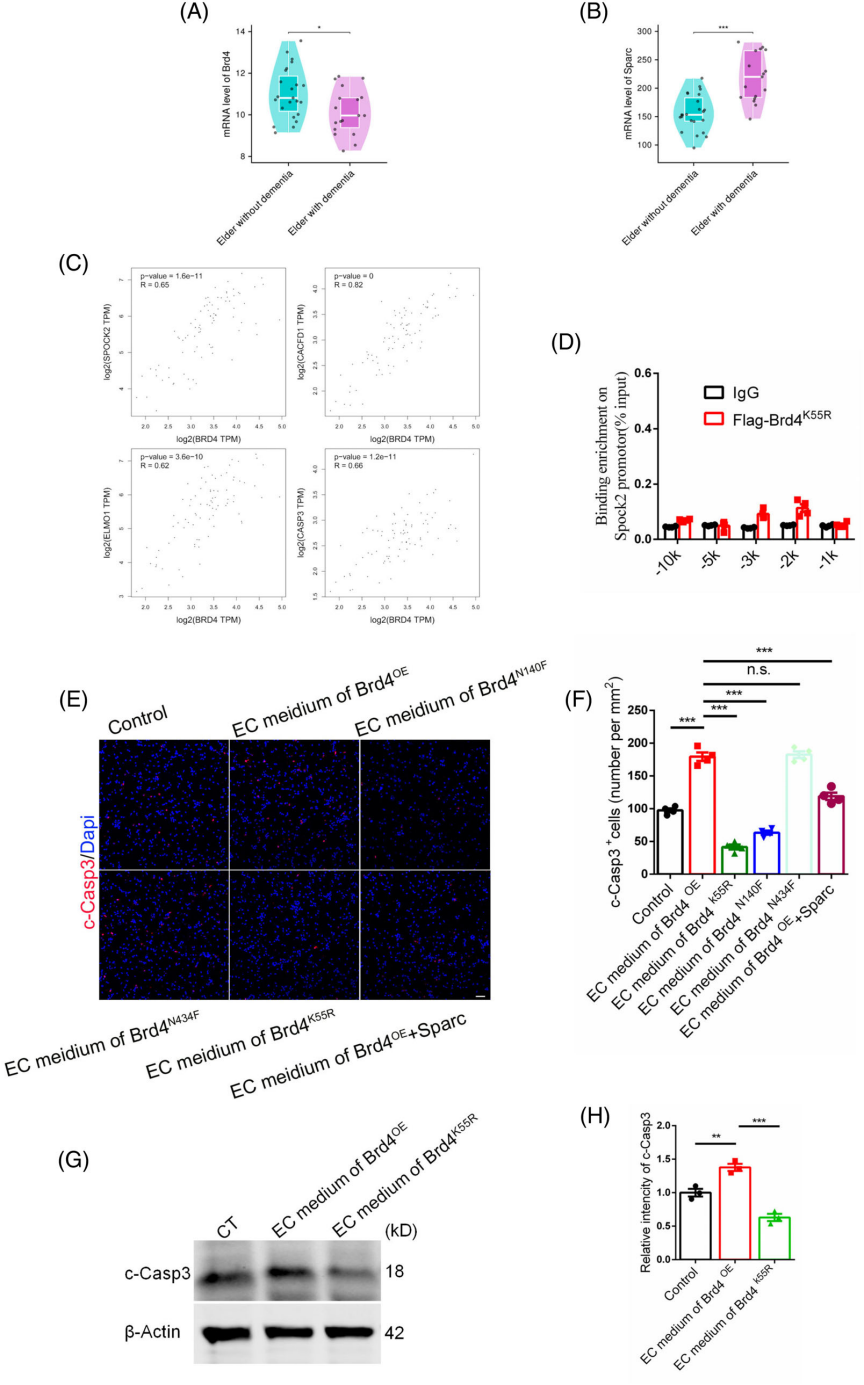
Brd4 is negatively correlated with dementia. (A) Comparison of the level of hippocampal Brd4 of elders with dementia (*n* = 18) and normal healthy elders (*n* = 23) (Data are from Allen Brain Atlas, and analysed using Wilcoxon Test). (B) Comparison of the level of hippocampal Sparc of elders with dementia (*n* = 18) and normal healthy elders (*n* = 23) (Data are from Allen Brain Atlas, and analysed using Wilcoxon Test). (C) The correlation between BRD4 and SPOCK2, CACFD1, ELMO1, CASP3. (D) Binding potential detection of Brd4 on the promotor of Spock2 after its 55th Lysine mutation, *n* = 4. (E) Immunofluorescent staining for c‐Casp3 with primary neural stem cells after add‐in endothelial cells medium. Scale bar, 50 μm. (F) Statistic analysis of c‐Casp3 positive cells, *n* = 4. (G) Proapoptotic effect of Brd4 and its mutant on neural stem cells. (H) Statistic analysis of relative intensity of c‐Casp3 in WB detection, *n* = 3.

Research indicates that cell communication is diminished in patients with Alzheimer's disease.^44^ It is of interest to investigate whether individuals with dementia exhibit a reduced state of cell competitiveness. By checking Allen Brain Map (https://portal.brain-map.org/), we found that patients with dementia exhibit decreased levels of the cell competitive positive regulator Brd4 (Figure 9A, Table S2) and increased levels of the cell competitive negative regulator Sparc (Figure 9B, Table S3) in the hippocampus. By checking genes interactive database (http://gepia.cancer-pku.cn/), we found a positively‐correlation between BRD4 and SPOCK2, CASP3, canonical cell competitive gene CACFD1 and engulfment related gene ELMO1 in hippocampus (Figure 9C). By checking DisGeNET database (https://www.disgenet.org/home/), it was observed that the 55th amino acid of protein Brd4 undergoes a mutation from lysine to arginine in Progressive supranuclear palsy patients. Utilising computational tools such as AlphaFold for structural prediction of protein Brd4, it was determined that this pathogenic mutational site interacts with two α‐helixes of domain BD1 (Figure S8A). This suggests a potential role of this amino acid in the formation of the hydrophobic pocket of BD1, with implications for the recognition and binding of BD1 to its downstream target. Stability analysis conducted using DUET indicated a decrease in the structural stability of protein Brd4 due to the mutation (Figure S8B). Furthermore, alignment of mouse and human Brd4 mRNA sequences revealed conservation of this site between the two species. In order to validate our hypothesis, we replicated the mouse Brd4 mutant and performed a CHIP assay to assess its interaction with the promoter of Spock2. The results indicated that the mutant significantly disrupted the binding between Brd4 and the promoter of Spock2 (Figure 9D). Subsequently, we evaluated its capacity for promoting cell competition in neural stem cells in vitro, which revealed a loss of such potential (Figure 9E–H). To consolidate the relationship between Brd4 and AD, we found another mutant Brd4^P1046H^ in a group of AD patients from AlzData (http://www.alzdata.org/). Because of its conservation between human and mouse (1049 in mouse), we cloned this mutant of mouse and conduct CHIP assay. Results showed that this mutant nearly abolished the binding between Brd4 and the promotor of Spock2 (Figure S8C). These results suggest that endothelial Brd4 exerts a negative regulatory effect on dementia by modulating the secretion of Testican2 through its BD1 domain, with Testican2 enhancing cell competition.

## 4 DISCUSSION

It is noteworthy that blood vessels are present in nearly all tumours and developing organs, its functions on non‐nutrition supplement got increasing attentions. Cell competition is positively correlated with tumour invasion. Although cell competition has been observed in developing organs, its role beyond determining cell death remains enigmatic. Drawing inspiration from the role of cell competition in tumour growth, we propose that blood vessels may play a role in the expansion of developing organs through a mechanism involving cell competition. Given its high expression in tumour vascular systems and its positive correlation with invasive genes, we suggest that Brd4 may serve as a link between blood vessel and cell competition. In our study, it was observed that the absence of endothelial Brd4 in mice led to the compromised neural stem cells competition through the action of the secreted protein Testican. Testican2 was found to function as a facilitator of competition among neural stem cells by reducing the levels of Sparc, thereby ensuring the elimination of less viable cells. In addition to its established role, cell competition was also discovered to influence the fate determination of neural stem cells, compromised cell competition resulting in a depletion of the stem cell pool and accelerated ageing. Interestingly, Testican2 was able to replenish the neural stem cell pool and enhance the turnover of neurons in a model of senile mice.

Cell competition has been implicated in the pathogenesis of dementia. Our investigation revealed a positive association between dementia and cell competitive negatively‐regulated protein Sparc, as well as a negative association between dementia and cell competitive positively‐regulated protein Brd4. Additionally, we identified a mutant form of Brd4 in Progressive supranuclear palsy patients, which attenuates the ability of Brd4 to promote cell competition by disrupting its interaction with the promoter of Spock2.

Building upon these experimental findings, we propose a novel therapeutic approach for age‐related diseases by enhancing the efficacy of cell competition. Intriguingly, Coelho et al. observed that controlled neuronal apoptosis conferred benefits when employing a drosophila AD model.^45^ In our assessment, the extent of their contribution was constrained. Primarily, the authors failed to adequately address the causal relationship between Aβ42 and losers, as they merely identified certain markers of losers in neurons expressing Aβ42 without effectively discerning whether Aβ42 produced losers or losers produced Aβ42, medicinal purposes targeting Aβ42 have been demonstrated are inefficient. Additionally, their principal downstream target Azot was not an original discovery, as its role in the clearance of suboptimal cells was not novel^10^; furthermore, despite the passage of 7 years since the initial discovery, research targeting Azot for medicinal purposes has not yielded any notable advancements. In contrast to previous research, our study identified alterations in cell competitive‐related genes in individuals with natural dementia. Through in vitro experimentation, we simulated a natural mutation of Brd4 in dementia patients and observed a decrease in the intensity of cell competition among neural stem cells, thereby implicating it in the pathogenesis of the disease. Additionally, we established a connection between brain blood vessels and the fate determination of neural stem cells through cell competition. Notably, our research uncovered a novel positive regulator of cell competition, Spock2, which has the potential to enhance the competitive intensity of cells and eliminate suboptimal cells in the treatment of age‐related diseases.

It is noteworthy that statins, a type of medication classified as lipid‐lowering drugs, have been found to reduce the occurrence of dementia regardless of its lipid‐lowering effects.^46^ Specifically, one particular statin, Atorvastatin has been shown to impede the progression of Alzheimer's disease.^47^ Our research has elucidated the underlying mechanism by which statins impact the treatment of ageing.

Furthermore, in the context of tumour research, blood vessels may play a crucial role in promoting the secretion of competitive factors that enhance competition between tumour and normal tissues. This dynamic may result in the survival of cancerous cells at the detriment of normal somatic cells. The majority of medications targeting blood vessels are currently directed towards angiogenesis in cancer therapy. It is hypothesised that targeting the secretory function of blood vessel may offer a more expedient approach to managing tumour invasiveness. Additionally, our research suggests that antagonising the BD1 domain of Brd4 may not be as innocuous as previously believed, as its delayed side effects, such as accelerated ageing or age‐related neurodegenerative diseases, may manifest over time.

While our study expands upon the notion of cell competition and its potential benefits for treating age‐related diseases, it is important to acknowledge that further refinement is necessary. For instance, the dynamic nature of cell competition was observed to impact the cell fate of neural stem cells, yet the actual process itself remains unobserved. Additional technologies, such as live imaging and cell tracing, are required to validate our hypothesis. Our findings suggest that enhanced cell competition may mitigate ageing, while increased cell deaths may not serve as a definitive indicator of strengthened cell competition. Furthermore, increased differentiation and decreased cell death were observed in instances of compromised cell competition, prompting inquiry into the relationship between cell death and differentiation. Future research endeavours are necessary to address these inquiries.

## AUTHOR CONTRIBUTIONS

Chenxiao Li, Jianwei Jiao, Jingjing Zhang and Fen Ji conceived the experiments. Mengtian Zhang and Yushan Du provided some helps in in vitro studies. Da Li and Shuang Liu provided some helps in the experiment performance. Shukui Zhang gave some advices in the draw of violin plot. J.J. supervised the project and obtained funding support.

## CONFLICT OF INTEREST STATEMENT

The authors declare no competing interests.

## Supporting information


**DATA S1:** Supporting Information.

## Data Availability

The data that support the findings of this study are openly available in Cell competition regulates the fate decision of neural stem cells at https://www.ncbi.nlm.nih.gov/geo/query/acc.cgi?acc=GSE223131, reference number GSE223131.
