# Supplementary material for: Compromised cell competition exhausts neural stem cells pool
Source: Cell Prolif. 2024 Jul 15;57(12):e13710. doi: 10.1111/cpr.13710 (PMC11628731; doi:10.1111/cpr.13710)

Supplementary Materials for

Compromised cell competition exhausts neural stem cells pool

Chenxiao Li et al.

Corresponding author: Jianwei Jiao, jwjiao@ioz.ac.cn

This PDF file includes:

Figures S1 to S8

Tables S1 to S4

Fig S1. Brd4 regulates neural stem cell deaths

(A) Single cell analysis of the expression of Brd4 in several cancers. (B) Carton model of the construct strategy of Brd4 flox mice. (C) WB detection for Brd4 with mice brain vascular endothelial cells at E14. (D) Statistic analysis of relative intensity of Brd4 in WB detection. n=4. (E) Immunofluorescent staining for Brd4 and Cd31 using primary endothelial cells. Scale bar, 10um. (F) Immunofluorescent staining for Brd4 and Ib4 with mice brains at P0. Scale bar, 50um. (G) Immunofluorescent staining for c-Casp3 with mice brains at P0. Scale bar, 50um. (H) Statistic analysis of c-Casp3 positive cells. n=4. (I) WB detection for c-Casp3 with primary neural stem cells after add-in of medium had cultured endothelial cells or not. (J) Statistic analysis of relative bond intensity of c-Casp3 in WB detection. n=4. (K) WB detection for c-Casp3 with primary neural stem cells after add-in of EC medium (Brd4 knock-down). (L) Statistic analysis of relative intensity of c-Casp3 in WB detection. n=4. (M) WB detection for Tnfα with mice cerebral cortex at E15. (N) Statistic analysis of relative intensity of Tnfα in WB detection. n=4.

Fig S2. Brd4 regulates the state of cell competition among neural stem cells

(A) Fwe3 detection of neural stem cells treated by endothelilal medium (Brd4 knock-down) using RT-PCR. n=3. (B) Neural stem cells with Fwe3-mCherry overexpression. (C) Relative mRNA level of Elmo1 with mice cerebral cotex at E14. n=4.

Fig S3. Brd4 regulates the thanscription of Spock2 by its BD1 domain

(A) Fwe3 detection by RT-PCR with primary neural stem cells after add-in of endothelial medium (Pltp, Nampt or Spock2 knock-down). n=4. (B) Detection of binding potential of Brd4 on the promotor of Spock2 after mutated its BD1 binding site. n=3. (C) Detection of binding potential of Brd4 to the promotor of Spock2 after mutated its BD2 binding site. n=3.

Fig S4. Effect of Atorvastatin on the stemness maintaining of neural stem cells

(A) RT-PCR analysis of Pax6 using neural stem cells after Atorvastatin treatment (100 nM). n=4. (B) WB analysis of Sox2 after Atorvastatin treatment. (C) Statistic analysis of relative intensity of Sox2 in WB detection. n=4.

Fig S5. Maintaining of neural stem cells niche needs cell competition

(A) Ki67 staining with mice brain at 5 weeks old. Scale bar, 50 um. (B) Statistic analysis of Ki67 positive cells. n=4.

Fig S6. Compromised cell competition accelerating aging

(A) Tbr2 staining with mice brain at 15 months old. Scale bar, 50um. (B) Statistic analysis of Tbr2 positive cells. n=4. (C) Analysis of the expression of Brd4 in mice hippocampus at the level of single

cell by Single Cell Portal. (D) WB detection for P21, Sox2 and Tuj1 with mice cerebral cortex at 15 months old. (E) Statistic analysis of relative intensity of P21, Sox2 and Tuj1 in WB detection. n=4.

Fig S7. Atorvastatin decreased the run-off of neural stem cells in senile mice models

(A) Effect of Atorvastatin (20 mg/kg) on the maintaining of neural stem cells in mice senile models by detecting the mRNA level of Pax6. n=3. (B) WB detection for Sox2 with cerebral cortex of mice seninle models. (C) Statistic analysis of relative intensity of Sox2 in WB detection. n=4. (D) WB detection for Sparc with cerebral cortex of mice seninle models. (E) Statistic analysis of relative intensity of Sparc in WB detection. n=4.

Fig S8. Structure prediction of Brd4

(A) Structure prediction of Brd4 using AlphaFold. (B) Structure stability prediction of Brd4 mutant. (C) Detection of binding potential of Brd4 to the promotor of Spock2 after its 1049th Proline muted into Histidine. n=3.

Table S1. Correlation between Brd4 and some invasive related genes (From TISCH)
Table S2. Hippocampus sample ID used for Brd4 expression contrast (From Allen Brain Atlas)
Table S3. Hippocampus sample ID used for Sparc expression contrast (From Allen Brain Atlas)
Table. S4 Primers used in this paper.

Table S1. Correlation between Brd4 and some invasive related genes (From TISCH)

Gene	Gene2	Dataset	Celltype(major-lineage)	correlation
Brd4	TUBB	PAAD_GSE111672	Myeloid	0.763
Brd4	TUBB	OV_GSE158722	Fibroblasts	0.616
Brd4	TUBB	NET_GSE140312	Endothelial	0.82
Brd4	TUBB	PAAD_GSE111672	Myeloid	0.763
Brd4	TUBB	OV_GSE158722	Fibroblasts	0.616
Brd4	TUBB	NET_GSE140312	Endothelial	0.82
Brd4	TUBA1B	CRC_GSE166555	All lineage	0.644
Brd4	TUBA1B	OV_GSE158722	Fibroblasts	0.635
Brd4	H2AFZ	NET_GSE140312	Fibroblasts	0.645
Brd4	H2AFZ	PRAD_GSE141445	All lineage	0.626
Brd4	STMN1	PAAD_GSE111672	Endothelial	0.671
Brd4	PPIA	OV_GSE158722	Fibroblasts	0.609
Brd4	ACTB	CRC_GSE166555	All lineage	0.688
Brd4	ACTB	OV_GSE158722	Fibroblasts	0.608
Brd4	PSMA2	PAAD_GSE111672	Endothelial	0.621
Brd4	TPI1	PAAD_GSE111672	Myeloid	0.73
Brd4	TPI1	NET_GSE140312	Endothelial	0.687
Brd4	TPI1	CRC_GSE166555	All lineage	0.624
Brd4	CFL1	OV_GSE158722	Fibroblasts	0.603

Brd4	HMGB1	CRC_GSE166555	All lineage	0.693
Brd4	SLC25A5	PRAD_GSE141445	All lineage	0.604
Brd4	YBX1	CRC_GSE166555	All lineage	0.641
Brd4	YBX1	PRAD_GSE141445	All lineage	0.614
Brd4	YBX1	NET_GSE140312	Endothelial	0.606
Brd4	ENO1	CRC_GSE166555	All lineage	0.667
Brd4	ENO1	OV_GSE158722	Fibroblasts	0.638
Brd4	SNRPD2	PAAD_GSE111672	Endothelial	0.76
Brd4	RAN	CRC_GSE166555	All lineage	0.607
Brd4	SNRPG	CRC_GSE166555	All lineage	0.638
Brd4	ANXA5	BCC_GSE141526	CD8T	0.64
Brd4	NEDD4	HB_GSE180665	All lineage	0.843
Brd4	TMEM212	LIHC_GSE146115	CD8T	0.659
Brd4	MYL6	PAAD_GSE111672	Myeloid	0.72
Brd4	PSMA7	CRC_GSE166555	All lineage	0.649
Brd4	PSMA7	PRAD_GSE141445	All lineage	0.607
Brd4	CENPF	LIHC_GSE146115	Myeloid	0.731
Brd4	RPL7	NET_GSE140312	Endothelial	0.807
Brd4	CCT5	CRC_GSE166555	All lineage	0.646
Brd4	ANXA2	NET_GSE140312	Endothelial	0.841
Brd4	ANXA2	OV_GSE158722	Fibroblasts	0.661

Table S2. Hippocampus sample ID used for Brd4 expression contrast (From Allen Brain Atlas)

People without dementia	People with dementia
496100319	496100332
496100339	496100338
496100349	496100365
496100353	496100372
496100355	496100388
496100384	496100396
496100389	496100417
496100406	496100433
496100471	496100470
496100477	496100487
496100480	496100503
496100490	496100509
496100491	496100519
496100536	496100539
496100547	496100597
496100570	496100635
496100573	496100664
496100576	496100666
496100586	

496100619	
496100622	
496100634	
496100654	

Table S3. Hippocampus sample ID used for Sparc expression contrast (From Allen Brain Atlas)

People without dementia	People with dementia
496100319	496100332
496100339	496100365
496100349	496100372
496100353	496100388
496100355	496100396
496100384	496100417
496100389	496100433
496100406	496100453
496100471	496100470
496100477	496100478
496100480	496100503
496100491	496100509
496100536	496100519
496100538	496100539
496100547	496100597
496100570	496100635
496100573	496100664
496100576	496100666
496100586	
496100619	
496100622	
496100634	
496100654	

Table S4. Primers used in this paper.

Brd4- shRNA-F	TGCCATCTACACTACGAGAGTTCTCGAGAACTCTCGTAGTGTAGATGGCTTTTTC
Brd4- shRNA-R	TCGAGAAAAAGCCATCTACACTACGAGAGTTCTCGAGAACTCTCGTAGTGTAGATGGCA
Spock2- shRNA-F	TCCATCGGTTGGATGTTCTCTACTCGAGTAGAGAACATCCAACCGATGGTTTTTC
Spock2- shRNA-R	TCGAGAAAAACCATCGGTTGGATGTTCTCTACTCGAGTAGAGAACATCCAACCGATGGA
Pltp- shRNA-F	TTGATCTCGTGGGCATCGATTACTCGAGTAATCGATGCCCACGAGATCATTTTTC

Pltp- shRNA-R	TCGAGAAAAATGATCTCGTGGGCATCGATTACTCGAGTAATC GATGCCCACGAGATCAA
Nampt- shRNA-F	TCCACCTTATCTTAGAGTCATTCTCGAGAATGACTCTAAGAT AAGGTGGTTTTTC
Nampt- shRNA-R	TCGAGAAAAACCACCTTATCTTAGAGTCATTCTCGAGAATG ACTCTAAGATAAGGTGGA
Brd4-identification-F	ACTGAGCTGCCATTGGGGTACATTA
Brd4-identification-F	ATGCTACCAGGTATTCCTAAACAAGATCT
Sparc-cag-F	ATGAGGGCCTGGATCTTC
Sparc-cag-R	GATCACCAGATCCTTGTTG
mFwe3-F	gcaGCGTTTAGCATGAG
mFwe3-R	gagGGAAATGGTGTTTCTG
pSicoR-F	TGTCAAAAAGGAAACTCACCC
pSicoR-R	GGCTATGAACTAATGACCCCGT
Brd4-aa140-pt-F	CAAATTGTTACATCTATttCAAGCCTGGAGATGACA
Brd4-aa140-pt-R	TGTCATCTCCAGGCTTGaaATAGATGTAACAATTTC
Brd4-aa434-pt-F	CCAACTGCTACAAGTACttCCCCCCTGACCATGAAG
Brd4-aa434-pt-R	CTTCATGGTCAGGGGGGaaGTACTTGTAGCAGTTGG
Brd4-aa55-pt-F	CCTCCAACCCTAACAgGCCCAAGAGACAG
Brd4-aa55-pt-R	cTGTTAGGGTTGGAGGTCTCTGGGGGT
Spock2-F	CGCGCTCCAGGCTCCGGGC
Spock2-R	CTACCAGATGTAGCCTCCATC
mFwe1-RT-F	TCCACACTTCTCTGGTTCTG
mFwe1-RT-R	GTGAGTACTGCTGTCTAGCC
mFwe2-RT-F	CGATGCCATTTCTTATGCTC
mFwe2-RT-R	TGACACTCAGTCTTCTCCAG
mFwe3-RT-F	CAAACACAGTAGCTGAGAAGG
mFwe3-RT-R	TAGAGGGAAATGGTGTTTCTG
mFwe4-RT-F	GTTTGCTAAATCCTGGGTGTC
mFwe4-RT-R	GCGTTCATGATCATCCACAC
Spock2-10k-F	TCCCACATGGGTAGGTGTGT
Spock2-10k-R	CCAGGAGAACCCTTACTACGC
Spock2-5k-F	AAACCTTCTTGGTGTGGGGG
Spock2-5k-R	CCTAAGCCTCACCTTGGCAT
Spock2-3k-F	TTCCCCATCCACTCACGTTC

Spock2-3k-R	AACAGCTGGTCCCTGTGTTG
Spock2-2k-F	TCTCCCTCAACACTAGCCGA
Spock2-2k-R	AAAGCAACCCAATGCCGTTC
Spock2-1k-F	CAGGCCCTGGATGACTGTAAC
Spock2-1k-R	AGACTACGAAAAGGGCAAACCA

Fig.S1

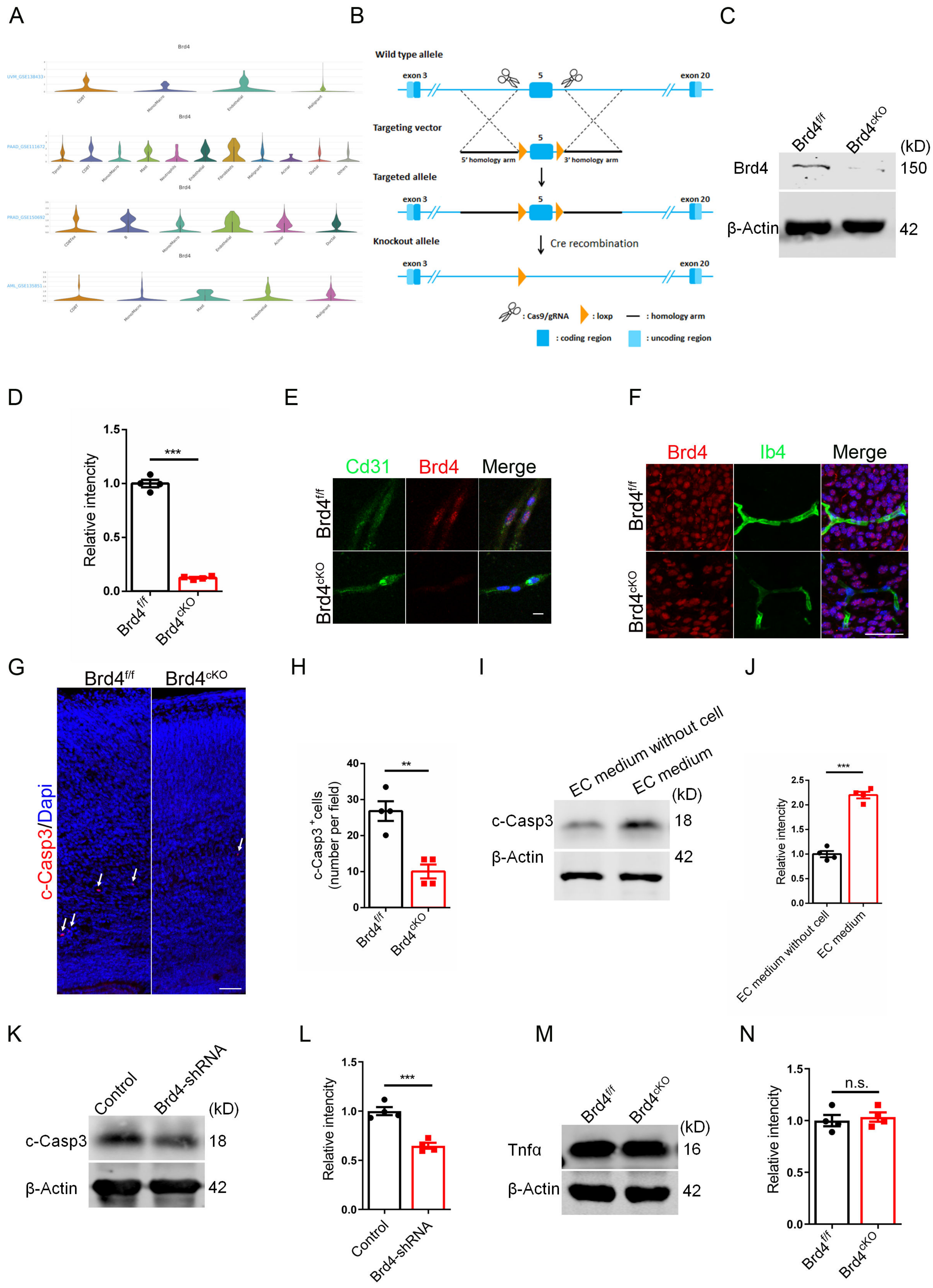
A
B
C
Wild type allele
exon 3
5
exon 20
Targeting vector
5' homology arm
3' homology arm
Targeted allele
Knockout allele
Cre recombination
: Cas9/gRNA
: loxp
: homology arm
: coding region
: uncoding region
Brd4f/f
Brd4cKO
(kD)
Brd4
150
β-Actin
42
D
E
F
Relative intencity
***
Cd31
Brd4
Merge
Ib4
G
H
I
J
c-Casp3/Dapi
c-Casp3+cells (number per field)
**
EC medium without cell
EC medium
c-Casp3
18
K
L
M
N
Control
Brd4-shRNA
Tnfα
16
n.s.

Fig.S2

A

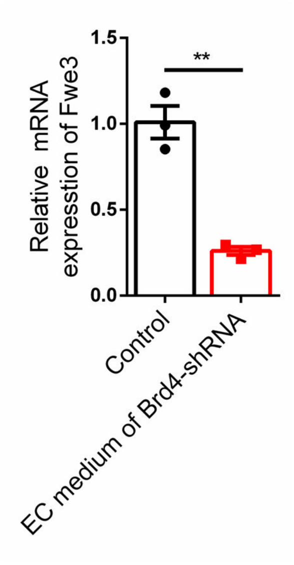

Relative mRNA expression of Fwe3
1.5
1.0
0.5
0.0
**
Control
EC medium of Brd4-shRNA

B

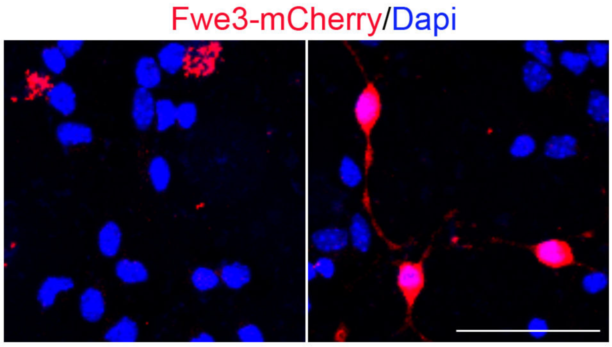

Fwe3-mCherry/Dapi

C

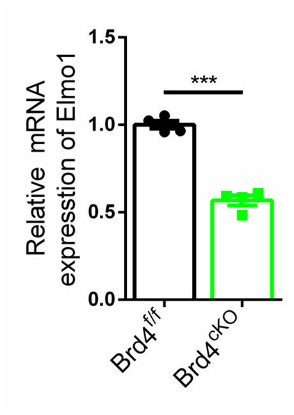

Relative mRNA expresstion of Elmo1
1.5
1.0
0.5
0.0
***
Brd4f/f
Brd4cKO

Fig.S3

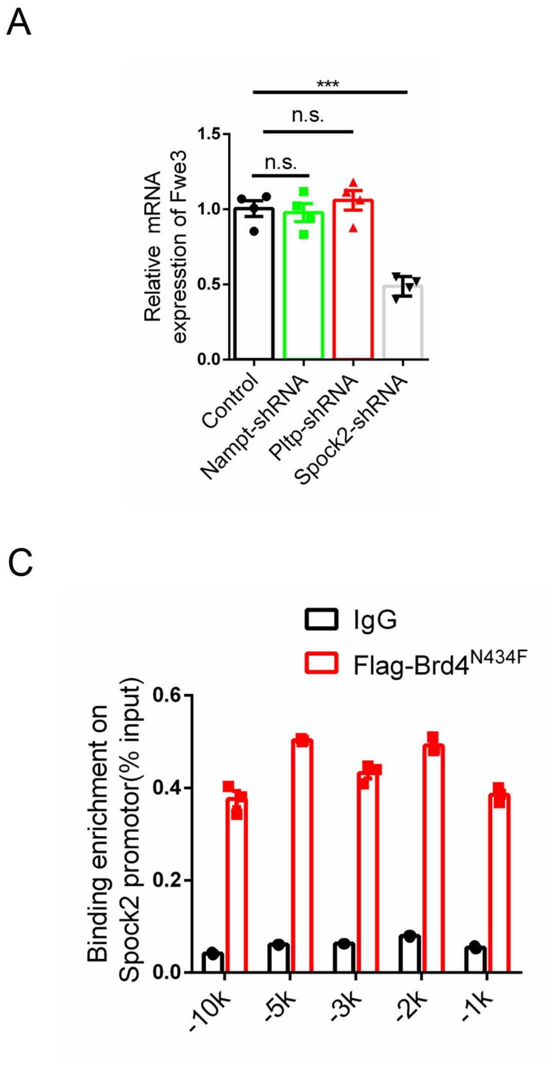
A
***
n.s.
n.s.
Relative mRNA expresstion of Fwe3
1.5
1.0
0.5
0.0
Control
Nampt-shRNA
Pltp-shRNA
Spock2-shRNA
B
Binding enrichment on Spock2 promotor(% input)
0.6
0.4
0.2
0.0
IgG
Flag-Brd4N140F
-10k
-5k
-3k
-2k
-1k
C
IgG
Flag-Brd4N434F
Binding enrichment on Spock2 promotor(% input)
0.6
0.4
0.2
0.0
-10k
-5k
-3k
-2k
-1k

Fig.S4

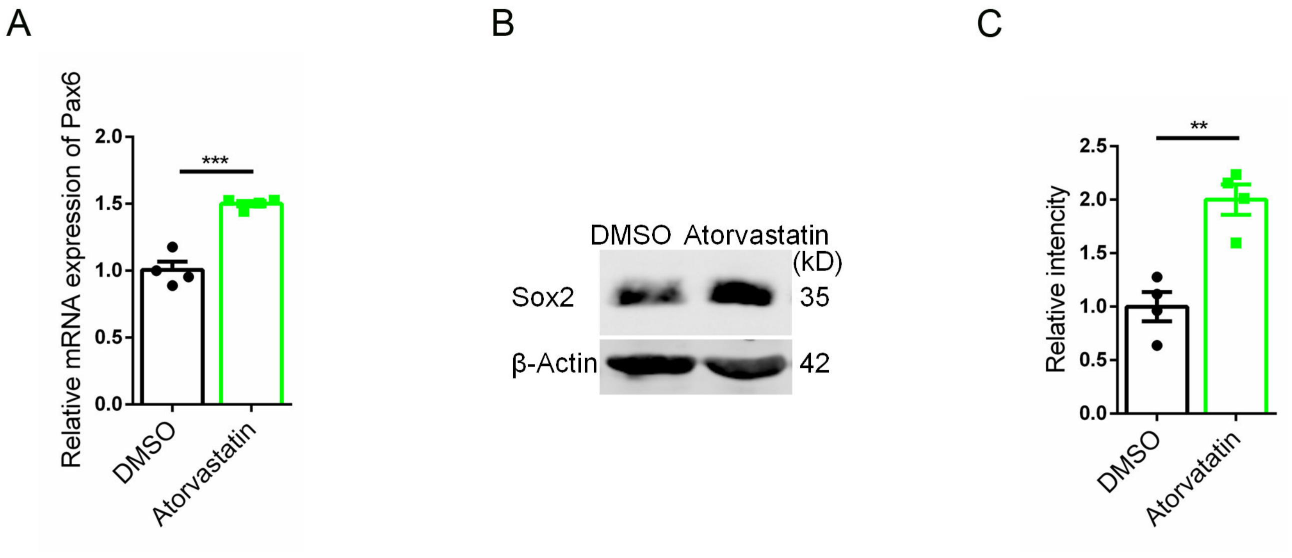
A
Relative mRNA expression of Pax6
2.0
1.5
1.0
0.5
0.0
***
DMSO
Atorvastatin
B
DMSO Atorvastatin
(kD)
Sox2
35
β-Actin
42
C
Relative intencity
2.5
2.0
1.5
1.0
0.5
0.0
**
DMSO
Atorvatatin

Fig.S5

A

B

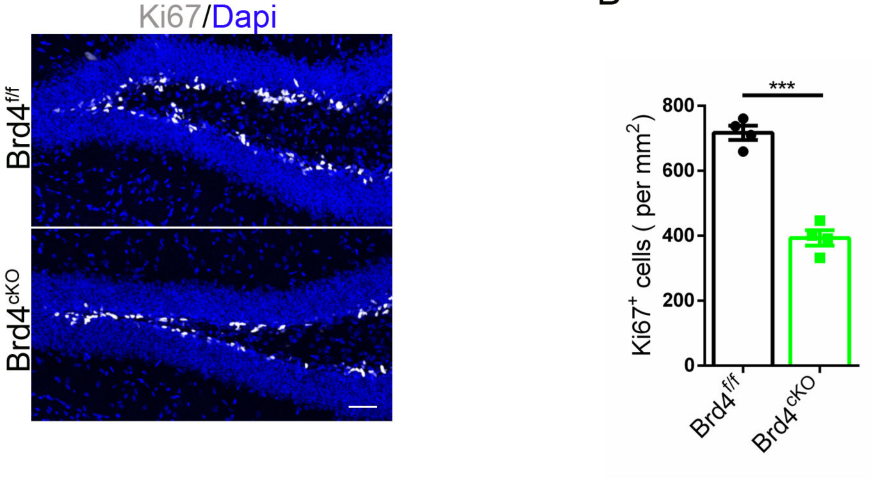
Ki67/Dapi
Brd4f/f
Brd4cKO
***
800
600
400
200
0
Ki67+ cells (per mm2)
Brd4f/f
Brd4cKO

Fig.S6

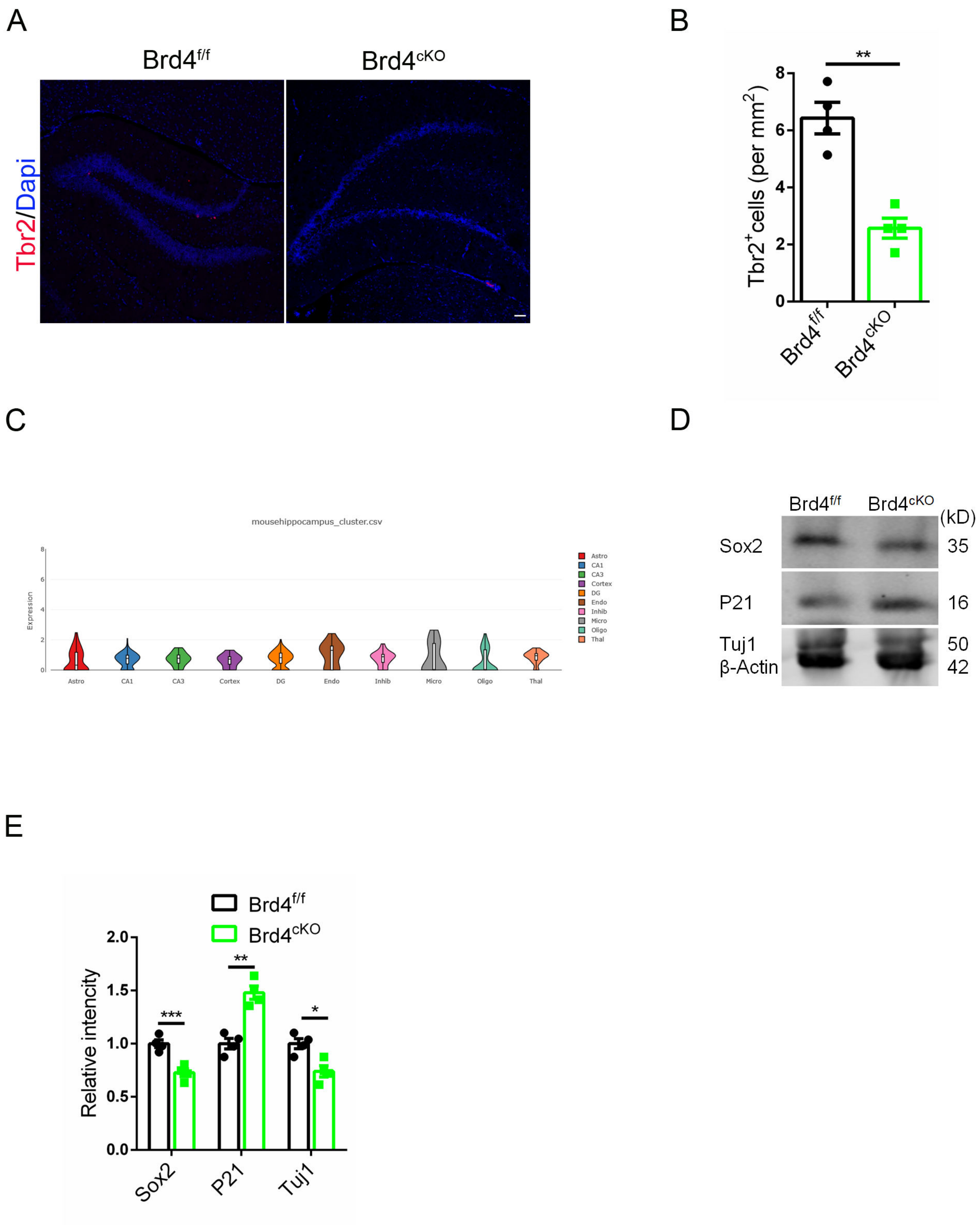
A
Brd4f/f
Brd4cKO
Tbr2/Dapi
B
**
Tbr2+cells (per mm2)
0
2
4
6
8
Brd4f/f
Brd4cKO
C
mousehippocampus_cluster.csv
Expression
Astro
CA1
CA3
Cortex
DG
Endo
Inhib
Micro
Oligo
Thal
D
Brd4f/f
Brd4cKO
(kD)
Sox2
35
P21
16
Tuj1
50
β-Actin
42
E
Brd4f/f
Brd4cKO
Relative intencity
0.0
0.5
1.0
1.5
2.0
***
**
*
Sox2
P21
Tuj1

Fig.S7

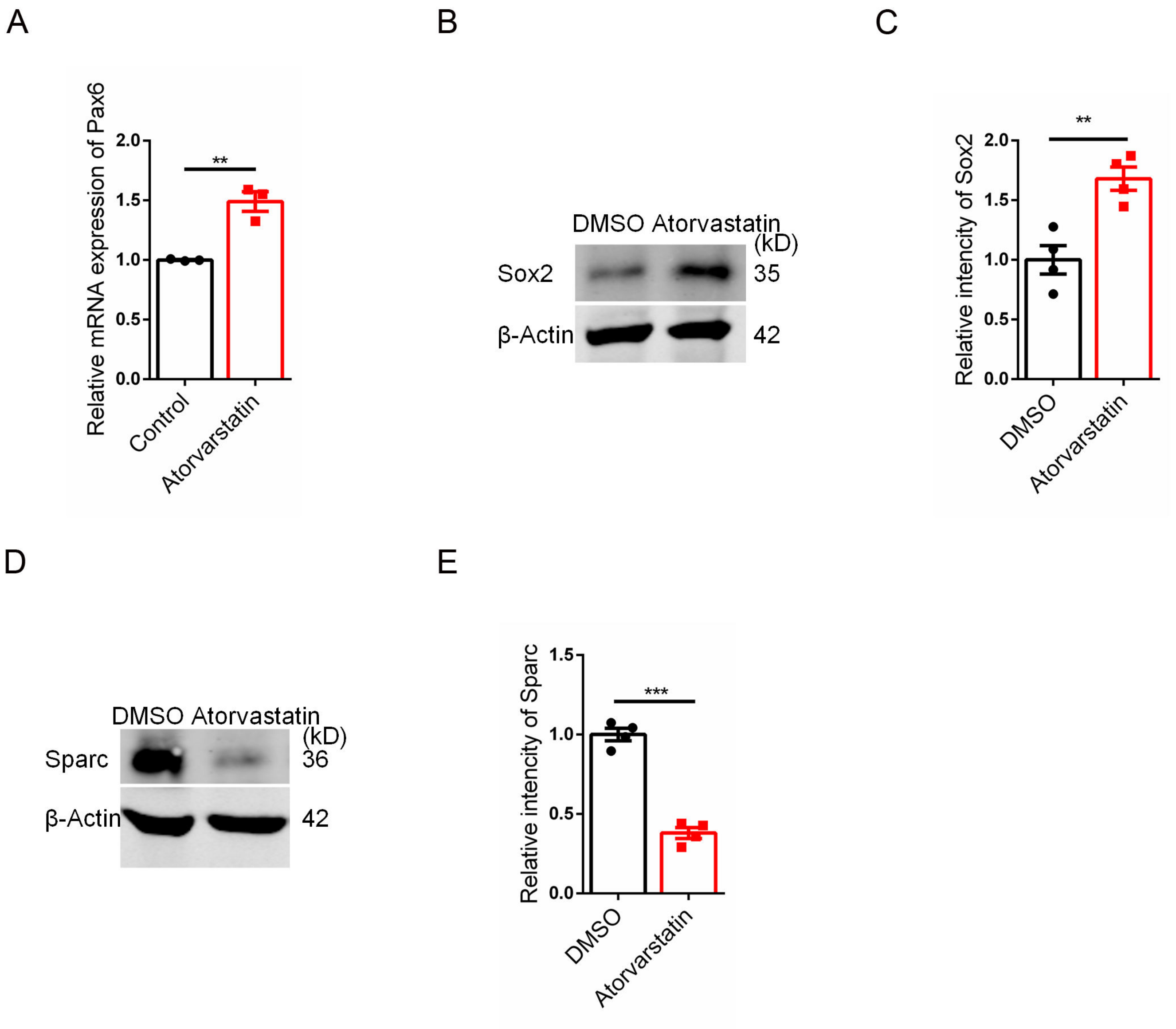
A
Relative mRNA expression of Pax6
2.0
1.5
1.0
0.5
0.0
**
Control
Atorvarstatin
B
DMSO Atorvastatin
(kD)
Sox2
35
β-Actin
42
C
Relative intencity of Sox2
2.0
1.5
1.0
0.5
0.0
**
DMSO
Atorvarstatin
D
DMSO Atorvastatin
(kD)
Sparc
36
β-Actin
42
E
Relative intencity of Sparc
1.5
1.0
0.5
0.0
***
DMSO
Atorvarstatin

Fig.S8

A

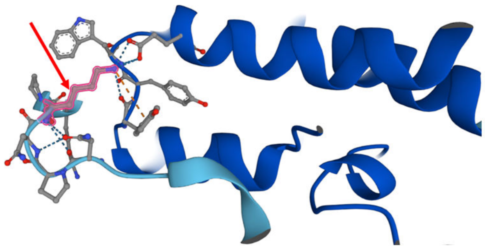

B

mCSM Predicted Stability Change (ΔΔG):

-0.72 kcal/mol (*Destabilizing*)

SDM Predicted Stability Change (ΔΔG):

0.1 kcal/mol (*Stabilizing*)

DUET Predicted Stability Change (ΔΔG):

-0.551 kcal/mol (*Destabilizing*)

Mutation:

Wild-type: **LYS**
Position: **55**
Mutant-type: **ARG**
Chain: **A**
Secondary structure: **Loop or irregular**

C

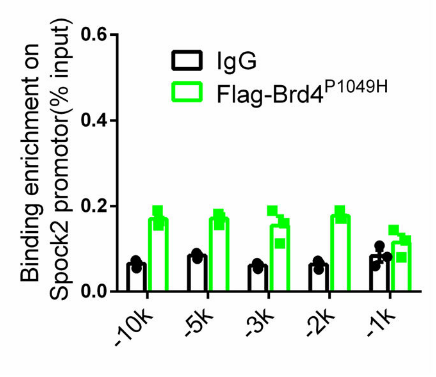

Supplement: Supplementary file 1 — DATA S1: Supporting Information. [file CPR-57-e13710-s001.pdf]
